# Force Field Effects in Simulations of Flexible Peptides with Varying Polyproline II Propensity

**DOI:** 10.1021/acs.jctc.1c00408

**Published:** 2021-09-15

**Authors:** Stéphanie Jephthah, Francesco Pesce, Kresten Lindorff-Larsen, Marie Skepö

**Affiliations:** † Division of Theoretical Chemistry, Lund University, SE-221 00 Lund, Sweden; ‡ Structural Biology and NMR Laboratory & the Linderstrøm-Lang Centre for Protein Science, Department of Biology, University of Copenhagen, DK-2200 Copenhagen, Denmark

## Abstract

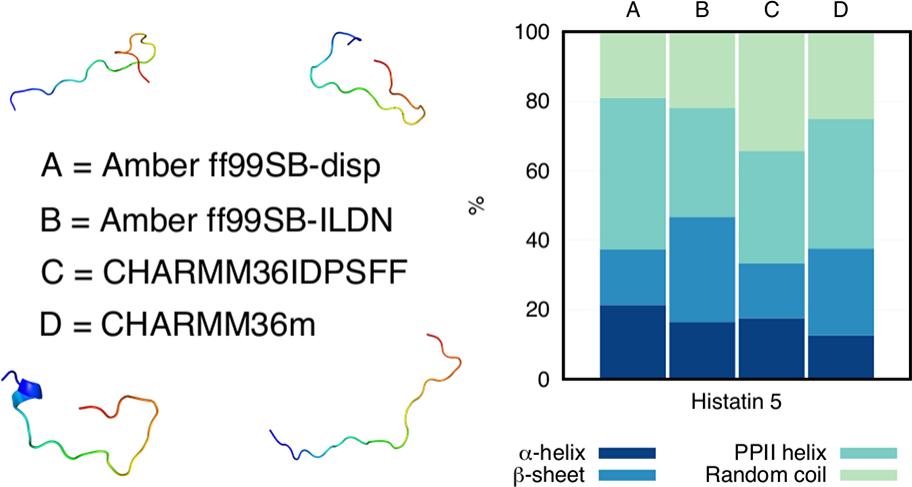

Five peptides previously suggested to possess polyproline II (PPII) structure have here been investigated by using atomistic molecular dynamics simulations to compare how well four different force fields known for simulating intrinsically disordered proteins relatively well (Amber ff99SB-disp, Amber ff99SB-ILDN, CHARM36IDPSFF, and CHARMM36m) can capture this secondary structure element. The results revealed that all force fields sample PPII structures but to different extents and with different propensities toward other secondary structure elements, in particular, the β-sheet and “random coils”. A cluster analysis of the simulations of histatin 5 also revealed that the conformational ensembles of the force fields are quite different. We compared the simulations to circular dichroism and nuclear magnetic resonance spectroscopy experiments and conclude that further experiments and methods for interpreting them are needed to assess the accuracy of force fields in determining PPII structure.

## 1 Introduction

Intrinsically disordered proteins and regions (IDPs and IDRs)—also recognized as natively unfolded proteins and peptides—are characterized by the lack of a well-defined tertiary structure in aqueous solution. Their structural properties are known to vary significantly, which makes it difficult to study them by standard methods. For example, because of their flexible behavior, IDPs and IDRs cannot be crystallized,^1^ and until relatively recently, many molecular simulations of flexible peptides and proteins had a strong bias for sampling α-helical and β-sheet structures as well as too compact and stable structures.^2−4^

Despite IDPs often being thought of as “unordered”, studies originating from the 1970s discovered that a few natively unfolded peptides possessed some degree of local order in their backbones, identified as the left-handed polyproline II (PPII) helix.^5^ Although the PPII helix was discovered a long time ago, it is still substantially less known than, for example, the α-helix and the β-sheet.^6^ One of the more recognized occurrences of the PPII helix might be as being part of the triple-helix structure of collagen, where it helps stabilize the collagen structure,^7^ and indeed specific efforts have been devoted to optimizing computational models for collagen.^8,9^ Another well-known and important property of the PPII helix has been observed in the binding to SH3 domains,^10^ where it facilitates and mediates protein–protein interactions.^11,12^

As a secondary structure element, the PPII helix is decidedly different from the α-helix and the β-sheet and perhaps less well-known, although frequently occurring in many proteins. The PPII helix has backbone dihedral angles of approximately (ϕ, ψ) = (−75°, +145°) with a helical pitch of 9.3 Å/turn and 3.0 residues/turn, which causes it to become quite extended.^6,13,14^ The peptide bond in a PPII helix is always found as the *trans* isomer (ω = +180°) since the *cis* isomer (ω = 0°) yields a different secondary structure known as the polyproline I (PPI) helix ((ϕ, ψ) = (−75°, +160°) with a helical pitch of 5.6 Å/turn and 3.3 residues/turn).^15^

One popular experimental technique for determining the secondary structure of proteins is circular dichroism (CD) spectroscopy. In the CD spectrum, the PPII helix is often associated with a strong band with negative ellipticity around 198 nm and a weak positive band around 218 nm.^5,6,16^ There are several software packages available for analyzing CD data and providing estimates of the relative secondary structure content, at least in terms of α-helices and β-sheets.^17−19^ Unfortunately, these algorithms may fall short when it comes to analyzing spectra of more disordered proteins that contain several less common secondary structure elements, including the PPII helix, and where the structural elements are not fixed in time. In such cases, the remaining secondary structure elements are lumped together and categorized as “others” or “random coils”. A similar problem is also encountered when the secondary structure content of protein structures is determined from simulations. Although the widely used DSSP (Dictionary of Secondary Structure of Proteins) program is able to identify and quantify a wider collection of secondary structure elements, it does not include the PPII helix. Fortunately, there is other software available that utilizes modified DSSP assignment to also include the PPII structure.^20,21^

The development of force fields for simulating IDPs is constantly evolving to help alleviate the problem of overly collapsed structures in simulations and to make the simulations as computationally efficient and accurate as possible.^2−4^ So far in force field development, the focus has mainly been on optimizing two aspects of IDP simulations.^22−25^ The first aspect is the secondary structure propensities, which are often modified by adjusting the protein backbone dihedral parameters.^26−32^ The second aspect concerns the balance of the protein–solvent interactions,^33,34^ which is crucial to not sampling too compact IDP conformations and to accurately capturing the more extended conformations. This is generally controlled by increasing and fine-tuning the interaction between the protein and the water in the simulations.

To evaluate new force fields for simulations of flexible peptides and proteins, different properties can be considered. Nuclear magnetic resonance (NMR) observables, such as scalar couplings and chemical shifts, are used for assessing force field accuracy by comparing simulated and experimental values and are in particular sensitive to local structural properties. Comparisons of scattering curves and the radius of gyration are used to evaluate the global compactness of the simulated proteins. In addition to such direct comparison to experimental observables, secondary structure propensities are often also assessed, and although more comprehensive analyses sometimes are used, they are most often restricted to the α-helix, the β-sheet, and the “random coil”. Conformational clustering is also sometimes used as a tool in force field analyses.

Here we present a study where five different short peptides (7–24 residues long) with varying PPII propensities, as well as five variants of one of the peptides, have been simulated with four different force fields that are known to work relatively well for simulating IDPs. Our analyses were mainly focused on differences regarding the secondary structure content across peptides and force fields. Our findings revealed that, although all the chosen force fields give rise to conformational ensembles with some level of PPII structure, they do so to different extents and with different propensities toward other secondary structure elements. Additionally, all force fields capture a trend showing that the PPII content increases with the number of Pro residues in peptide chains consisting of only Ala and Pro residues.

## 2 Methods

### 2.1 Molecular Dynamics Simulations

Five different peptides known to possess PPII structure, as well as five different variants of one of the peptides, were simulated using atomistic molecular dynamics (MD) simulations. Names and amino acid sequences of the selected peptides are shown in Table 1.

**Table 1:**
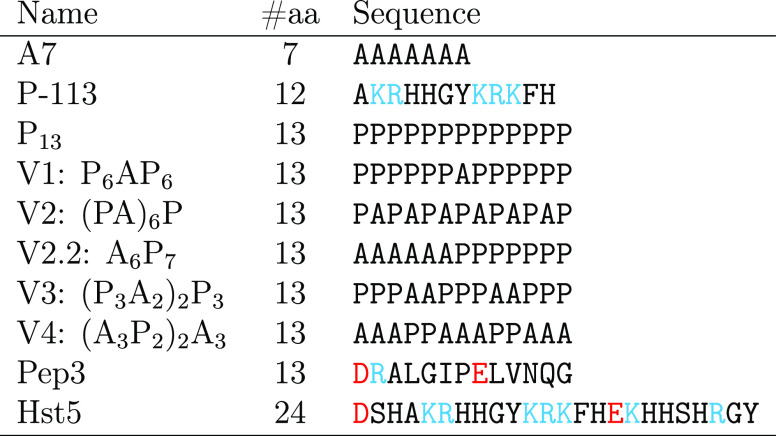
Names, Number of Amino Acid Residues (#aa), and Amino Acid Sequences of the Peptides Used in This Study^a^

a Positively charged amino acids are shown in blue, and negatively charged amino acids are shown in red.

The simulations were performed with the GROMACS package (ver. 4.6.7),^35−37^ with four different force fields that have also previously been used for simulating IDPs: (A) the AMBER ff99SB-disp force field with its own TIP4P-D-type water model,^38^ (B) the AMBER ff99SB-ILDN force field^28^ with the TIP4P-D water model,^34^ (C) the CHARMM36IDPSFF^39,40^ force field with the CHARMM-modified TIP3P water model,^41^ and (D) the CHARMM36m force field,^31^ also with the CHARMM-modified TIP3P water model. When discussed in the text, the force fields are referred to by their abbreviations, and when described in the figures and tables, they are referred to by their one-letter code (see Table 2). A rhombic dodecahedron was used as a simulation box, with periodic boundary conditions in all directions. A minimum distance of 1 nm was set between the solute and the box edges. The initial, linear protein structures were built with the use of PyMOL.^42^

**Table 2:** Force Field Notations

force field	abbreviation	one-letter code
AMBER ff99SB-disp	A99SB-disp	A
AMBER ff99SB-ILDN	A99SB-ILDN	B
CHARMM36IDPSFF	C36IDPSFF	C
CHARMM36m	C36m	D

The Verlet leapfrog algorithm,^43^ with a time step of 2 fs, was used to integrate the equations of motion. Nonbonded interactions were computed with a Verlet list cutoff scheme, short-ranged interactions were calculated by using a pair list with a cutoff of 1 nm, and long-ranged electrostatics were evaluated by using particle mesh Ewald summation^44^ with cubic interpolation and a grid spacing of 0.16 nm. Long-ranged dispersion interactions were applied to the energies and pressures of the simulated systems. All bond lengths were constrained by using the LINCS algorithm.^45^ A velocity-rescaling thermostat^46^ with a relaxation time of 0.1 ps was used to keep a temperature of 293 or 300 K (see Table 3 for details), and a Parrinello–Rahman barostat^47^ kept the pressure at 1 bar throughout the simulations. A relaxation time of 2 ps was used, and the isothermal compressibility was set to that of water, i.e., 4.5 × 10^–5^ bar^–1^.

**Table 3:** Force Fields (FF), Total Production Run Times (*t*), and Temperatures (*T*) of All the Simulations

peptide(s)	FF	*t* (μs)	*T* (K)
A7, P-113, P_13_, V1, V2, V3, V4, Pep3	A	5	300
A7, P_13_, V1, V2, V2.2, V3, V4, Pep3	B	5	300
A7, P-113, P_13_, V1, V2, V3, V4, Pep3	C	5	300
A7, P-113, P_13_, V1, V2, V3, V4, Pep3	D	5	300
P-113	B	12	300
Hst5	A	5	293
Hst5	B	7	293
Hst5	C	5	293
Hst5	D	5	293

Energy minimization was done by using the steepest descent algorithm. Equilibration of the temperature and pressure was done in two steps and with position restraints on the proteins: (1) 500 ps in the *NVT* ensemble and (2) 1000 ps in the *NPT* ensemble. Five replicates with different starting seeds were used for each simulation. The final production runs were performed in the *NPT* ensemble for a total of 5 μs (5 × 1 μs) for the majority of the peptides. Hst5 with A99SB-ILDN was run for a total of 7 μs (1 × 3 μs + 2 × 2 μs), and P-113 with A99SB-ILDN was run for a total of 12 μs (1 × 8 μs + 2 × 2 μs). The differences among all the simulations are summarized in Table 3. Simulation data of Hst5 with A99SB-ILDN and Hst5 with C36m have previously been published in the paper by Jephthah et al.^48^

### 2.2 Simulation Analyses

The GROMACS tool g_analyze was used to obtain averages, autocorrelation functions, and error estimates (block averaging)^49^ of the radius of gyration and the end-to-end distance of the simulated peptides. Results from these analyses were used to assess convergence and are presented in the Supporting Information (Figures S1–S15). The GROMACS tool g_cluster was used with the GROMOS algorithm^50^ to obtain conformational clusters, and to obtain frames for representative structures. All protein structures were visualized and rendered with PyMOL.^42^

#### 2.2.1 Principal Component Analysis

Principal component analysis (PCA) is a dimensionality reduction method that makes it possible to represent a fraction of the information contained in a large set of variables (or features) in a smaller set. This is achieved by calculating the eigenvectors (or principal components) of the variables’ covariance matrix. A PCA was performed, for each peptide, on an aggregated trajectory made by concatenating the trajectories resulting from the four different force fields. This, as suggested in ref (51), ensures a robust comparison of the force fields by projecting the resulting trajectories onto common principal components. PCA calculations were performed with pyEMMA^52^ using as features the cosine and sine of each backbone dihedral. The analyses were based on the first two principal components.

#### 2.2.2 Secondary Structure Analysis

The secondary structure was analyzed by using three methods: (i) the DSSP algorithm,^53^ (ii) the DSSP-PPII algorithm,^20^ and (iii) estimations from the Ramachandran plot. The last method is described in the following paragraph. The GROMACS tool g_chi was used to check the ω angles in the simulations (see Figure S16).

**Figure 1:**
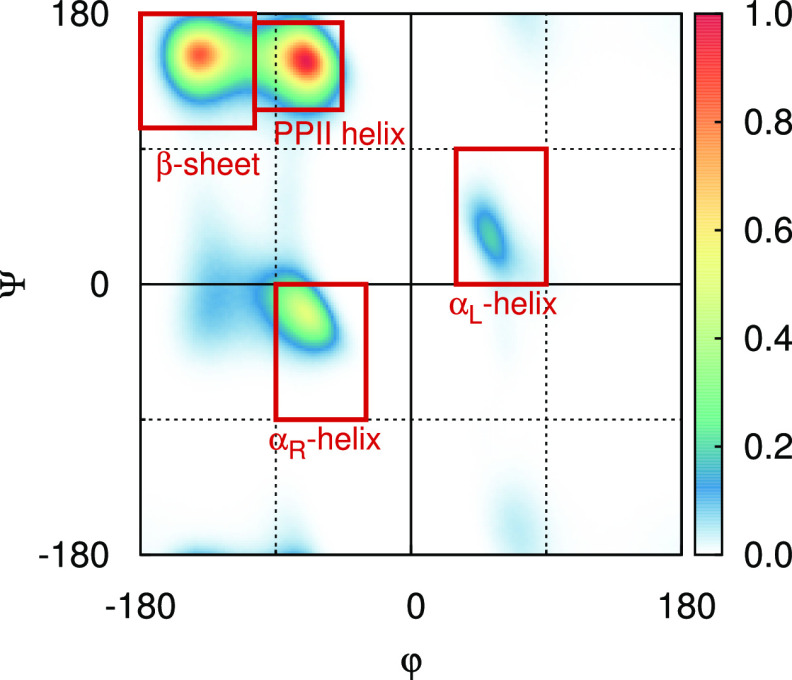
Example of a Ramachandran map illustrating the four different secondary structure regions analyzed in this study. The β-sheet region is defined by −180° ≤ ϕ < −104° and 180° ≤ ψ ≤ 104°, the PPII helix region is defined by −104° ≤ ϕ ≤ −46° and 116° ≤ ψ ≤ 174°, the α_R_-helix region is defined by −90° ≤ ϕ ≤ −30° and −90° ≤ ψ ≤ 0°, and the α_L_-helix region is defined by 30° ≤ ϕ ≤ 90° and 0° ≤ ψ ≤ 90°. Anything outside of these regions is classified as “random coil”. The plot is normalized for a maximum intensity of 1. The displayed angles of the example were obtained from simulations of Hst5 with A99SB-ILDN.

The local structural preferences were also estimated from the dihedral angles of the peptide backbones, which were obtained by using the GROMACS tool g_rama. Only the α-helical (both the right-handed and the left-handed), the β-sheet, and the PPII helical regions of the Ramachandran map were considered for this analysis. Similar to what has previously been done in many other studies,^9,20,26,27,30,31,54−58^ a residue was considered to be in the right-handed α-helix (α_R_) region of the Ramachandran map when −90° ≤ ϕ ≤ −30° and −90° ≤ ψ ≤ 0°, as illustrated in the Ramachandran map in Figure 1. Correspondingly, 30° ≤ ϕ ≤ 90° and 0° ≤ ψ ≤ 90° were used for the left-handed α-helix (α_L_) region, −180° ≤ ϕ < −104° and 180° ≤ ψ ≤ 104° were used for the β-sheet region, and −104° ≤ ϕ ≤ −46° and 116° ≤ ψ ≤ 174° were used for the PPII helix region. Residues not belonging to any of the aforementioned regions were unclassified but categorized as “bend/coil/turn” for simplification of the plots. We note that this classification is not based on secondary structure elements, but simply examines which regions of the Ramachandran map the different residues populate.

#### 2.2.3 CD Prediction

To predict CD spectra from structural ensembles, we employed SESCA.^59^ The SESCA algorithm has two steps:

1. The first step is per residue secondary structure assignment. We use DISICL^60^ as the secondary structure prediction algorithm as it explicitly takes into account PPII conformations.

2. Spectral contributions from each secondary structure element in a conformation are combined to produce the CD spectra. In SESCA, the set of spectral contributions assigned to subsets of secondary structures are stored in the “basis sets”. Different basis sets for a given secondary structure assignment are available, which differ in the resolution of the spectral contributions definition. Optionally, the spectral contributions of the side chains may be added. We tested several of the available basis sets, but mainly used DS6-1SC1 (DS6-1 with side chain contribution), as this gave rise to the predicted spectra that resembled the most in shape the experimental spectra.

Finally, the CD spectra from each conformation of the ensemble are linearly averaged.

### 2.3 Calculation of *J*-Couplings

We used Karplus-like equations^61^ to calculate the backbone NMR scalar (*J*) couplings. This equation has the functional form
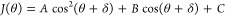
1

In eq 1, θ is the torsional angle that determines the *J*-coupling constant, while *A*, *B*, and *C* are fitting parameters and δ is a shift used in some calculations. For ^3^*J*_HNH_α__, ^3^*J*_H_α_C′_, ^3^*J*_HNC′_, and ^3^*J*_HNC_β__, we used the parametrization from Lindorff-Larsen et al.^62^ The same parametrizations employed in the work of Graf et al.^63^ were used for ^1^*J*_NC_α__,^64^^2^*J*_NC_α__,^65^ and ^3^*J*_HNC_α__.^66^

To compare the calculated coupling constants with the experimental values we used
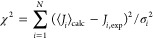
2where ⟨*J*_*i*_⟩_calc_ is the time average of the *i*th *J*-coupling constant from the frames of a simulation, *J*_*i*,exp_ is the respective experimentally observed *J*-coupling constant, and σ_*i*_ is the error associated with the parametrization of the Karplus relationship.^65−67^ Experimental errors and the errors on simulated averages are smaller than those from the Karplus parametrizations and were thus not included.

## 3 Results and Discussion

### 3.1 Effects of Force Field in Simulations of Five Peptides

An initial comparison of the effects of the force fields was done by analyzing the resulting average radius of gyration of the five peptides (Table 4). All force fields resulted in similar average values for the radius of gyration for each of the individual peptides, although C36IDPSFF on average resulted in slightly more compact conformations compared to the other force fields. This was, however, not the case for P_13_, for which C36m sampled a slightly smaller average instead. Overall, it seemed like both A99SB-disp and A99SB-ILDN sampled similar averages for all peptides.

**Table 4:** Radius of Gyration, *R*_g_ (nm), for the Five Peptides with the Four Different Force Fields

FF	A7^a^	P-113	P_13_^a^	Pep3	Hst5
A	0.62	0.97 ± 0.01	1.14	0.91 ± 0.03	1.29 ± 0.08
B	0.63	0.91 ± 0.03	1.14	0.94 ± 0.01	1.29 ± 0.05
C	0.60	0.86 ± 0.01	1.13	0.87 ± 0.01	1.18 ± 0.02
D	0.61	0.92 ± 0.02	1.11	0.97 ± 0.01	1.35 ± 0.03

a The values of A7 and P_13_ are reported without error margins because their errors are smaller than 0.005 nm.

**Figure 2:**
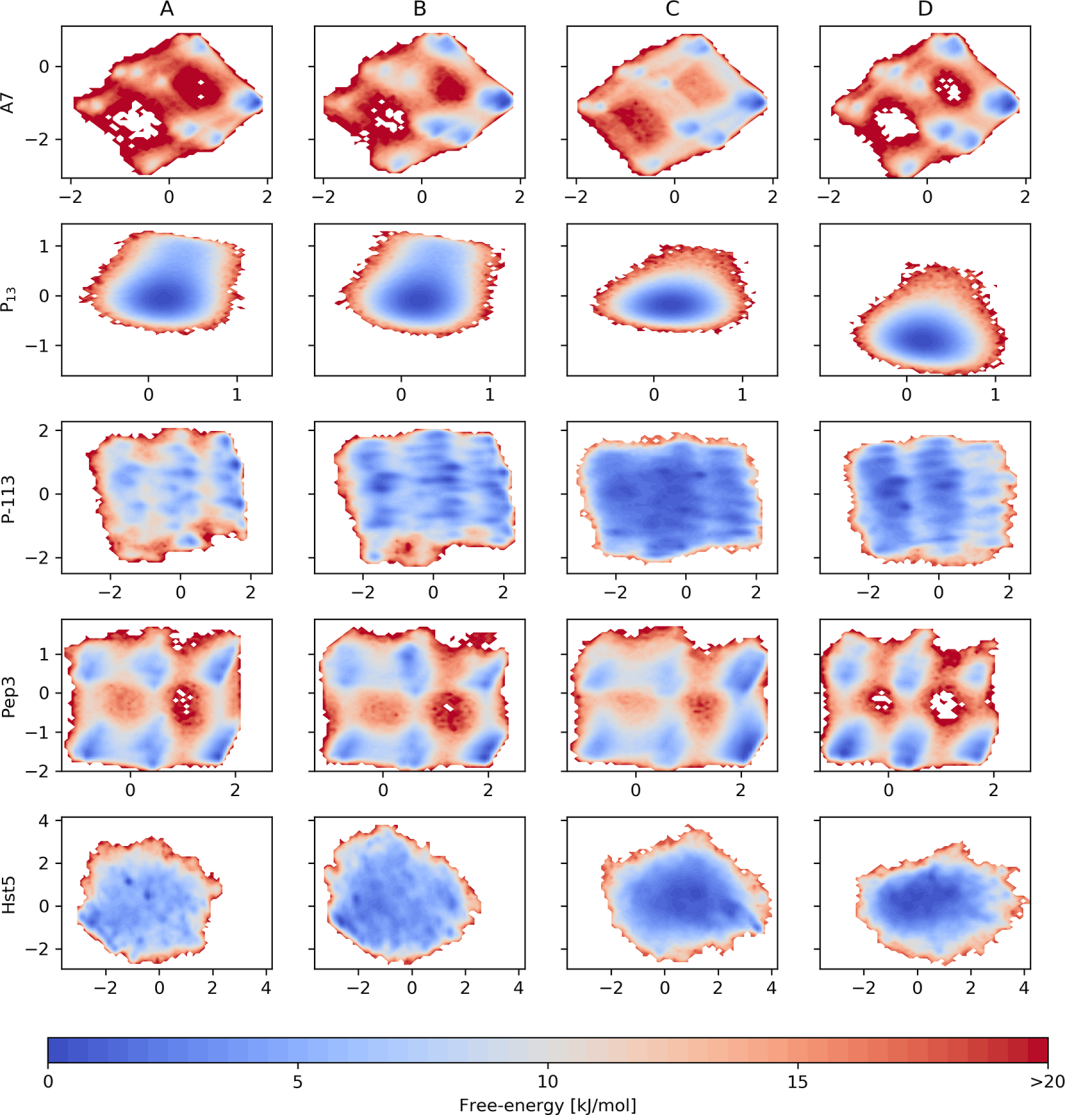
Free energy surfaces as a function of the first (*x*-axis) and second (*y*-axis) principal components. As a result of performing the PCA on the aggregated trajectories, the PC coordinates for all the force fields on a row are the same.

Because the conformational ensembles of IDPs are highly heterogeneous, it is not trivial to find a set of variables that can describe the high variability of an ensemble in a low-dimensional representation. For each peptide, we used PCA (on aggregated trajectories over all force fields as discussed under Methods) to represent and visualize the simulations in a space of reduced dimensionality. After projecting the ensembles from the different force fields onto a common subspace, we examine the free energy surfaces projected as a function of the first two principal components, and in general we find relatively similar surfaces. However, the relative probabilities of the conformational states may differ, with C36IDPSFF giving rise to less “rough” surfaces, while the others show regions poorly explored at the simulated temperature (Figure 2).

**Figure 3:**
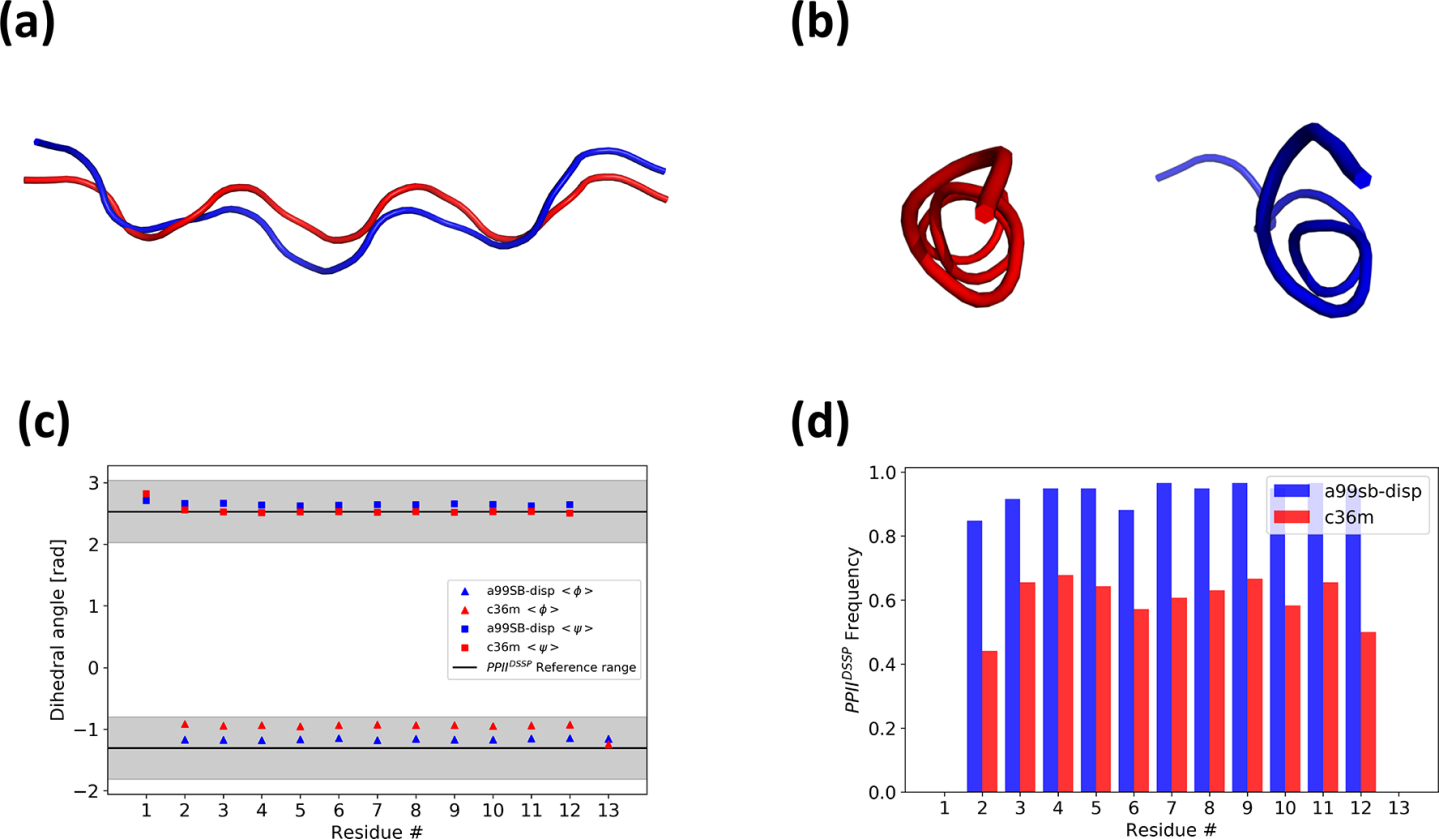
Structural analysis of free energy minima in A99SB-disp (blue) and C36m (red). (a) Side view and (b) C-terminal view of two representative P_13_ structures from A99SB-disp and C36m. (c) Average per residues ϕ and ψ dihedrals, compared to the PPII helix range defined in DSSP-PPII. (d) Per residue probability of PPII conformations as assigned by DSSP-PPII.

Nonetheless, it is worth highlighting that, in the case of P_13_ with C36m, we observe a shift of the minimum on the second PC axis. Since P_13_ is thought to mostly populate PPII conformations, we decided to characterize and compare the free energy minima resulting from A99SB-disp and C36m. Subtle differences were observed, both in the average radius of gyration (1.14 nm for A99SB-disp and 1.11 nm for CHARMM36m) and in the per residue average backbone dihedrals that, for both force fields, reside in the PPII ranges defined in DSSP-PPII^20^ (Figure 3c). At the level of local structure, we find that A99SB-disp populates more PPII conformations than C36m (Figure 3d). Additionally, the PPII helix formed in simulations with A99SB-disp appears more bent with respect to an imaginary helix axis, while the PPII helix formed with C36m appears to be straighter (Figure 3a,b).

**Figure 4:**
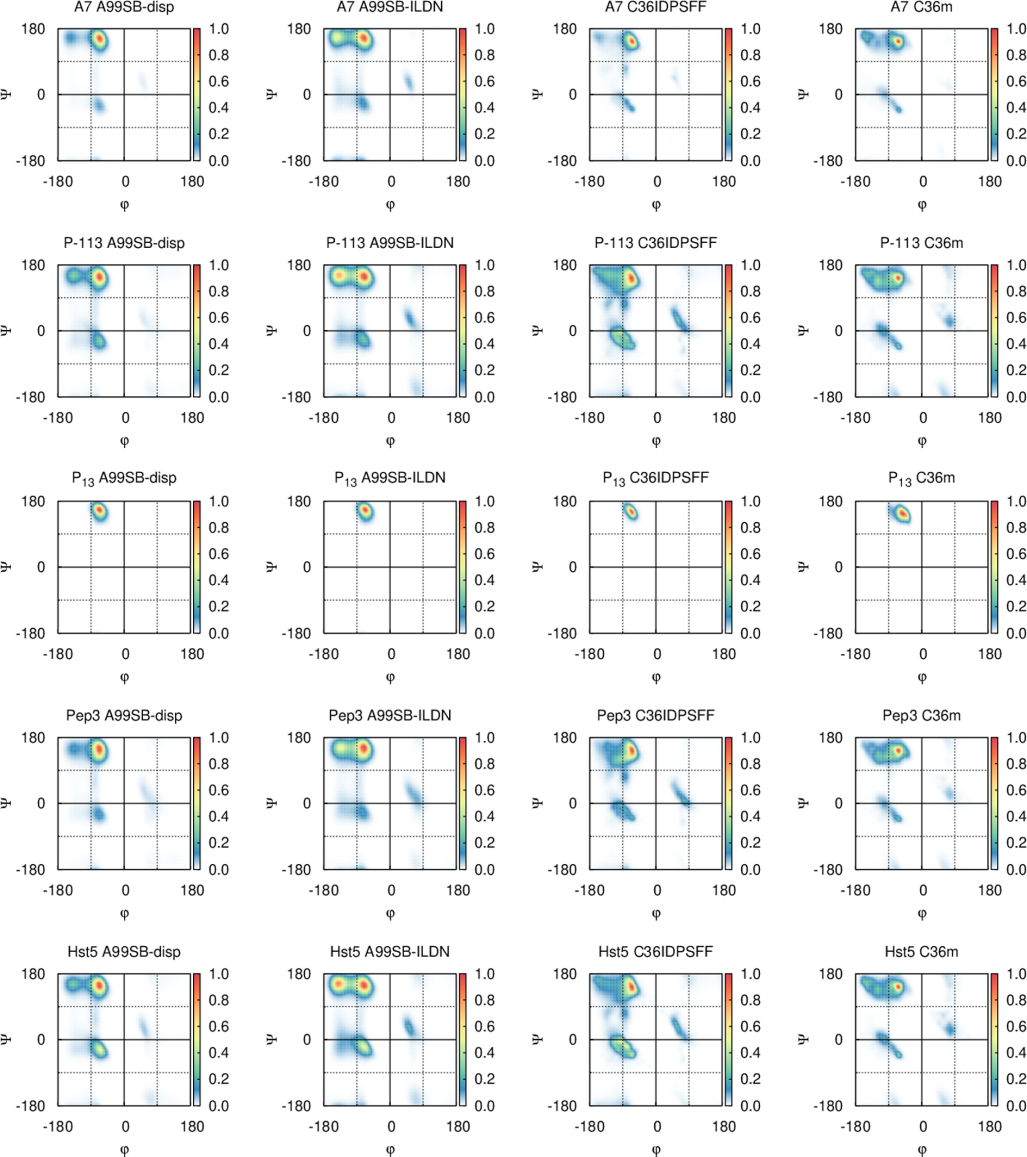
Ramachandran plots aggregated over the full sequence of the five main peptides from simulations using four different force fields. The plots are normalized for a maximum intensity of 1.

**Figure 5:**
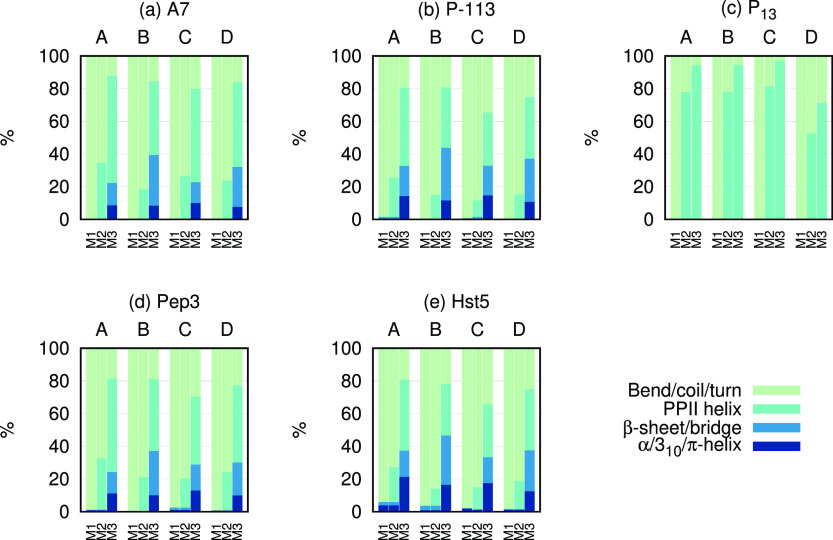
Stacked histograms of average secondary structure of simulated peptides obtained from three different methods: M1 = DSSP, M2 = DSSP-PPII, and M3 = Ramachandran populations of the regions defined in Figure 1. We note that while M1 and M2 refer to assessments of secondary structure, M3 only reports on the sampling of regions of the Ramachandran map of each individual residue independent of the conformations of its neighbors.

Ramachandran plots depicting all dihedral angles of each simulated peptide are presented in Figure 4. A few differences were observed when the force fields were compared. First, the Amber force fields (especially A99SB-ILDN) clearly show a more distinct β-sheet region and the populations in both the α-helix region and the β-sheet region seem to be confined to smaller and more concentrated regions in the Amber simulations compared to the CHARMM simulations, where they seem to be spread out over larger areas. It is also worth noting that all Amber simulations have similar appearances/distributions over the Ramachandran space. The same is observed for the CHARMM simulations, and their appearance/distribution is slightly different from that of the Amber simulations. This strongly suggests that the different force field families (as might be expected) sample different structures. Interestingly, the PPII region seems to be somewhat shifted in the case of P_13_ with C36m, which is not seen for the other peptide simulations with the same force field. From studying these aggregated distributions across the Ramachandran plots alone, it is nearly impossible to obtain any detailed information on secondary structure propensities. Thus, the region populations have been quantified and are presented in Figure 5, where they are also compared to secondary structure estimates from the DSSP and the DSSP-PPII methods.

Comparisons of the average secondary structure content of each simulated peptide using three different methods are depicted in Figure 5. The DSSP analysis suggested that all peptides were fully disordered and dominated by bends, coils, and turns. Further investigation using DSSP-PPII did, however, reveal that approximately 10–35% of the secondary structure content was, in fact, PPII structure for most of the peptides. The P_13_ peptide was found to possess even more PPII structure (∼50–80%), which is reasonable since it is expected to mainly possess PPII structure in aqueous solution.^68,69^

By comparing the secondary structure contents obtained from the different force fields using the DSSP-PPII method, A99SB-disp was found to sample more PPII structure than the other force fields for all peptides (except P_13_) and A99SB-disp and C36IDPSFF sampled slightly more α/3_10_/π-helical and β-sheet/bridge content. A7 and P_13_ possessed no other secondary structure elements according to this analysis. The amount of unstructured content (coil/bend/turn) was highest for C36m in the case of P_13_. No other obvious secondary structure propensities and trends were discerned.

Average populations of different regions of the Ramachandran map (corresponding to typical dihedral angles in different secondary structure elements) were also estimated from all of the simulations. Although this method was able to identify angles corresponding to PPII, it also showed large populations in the α-helical and β-sheet regions, which were not observed in the DSSP and the DSSP-PPII analyses. This is not surprising since the Ramachandran map includes all angles regardless of position, whereas secondary structures need several consecutive amino acids with the same classification for them to register as a secondary structure. The Ramachandran analysis indicated that C36IDPSFF provided less sampling in the structured regions for all peptides except P_13_, whereas A99SB-ILDN sampled larger populations in the β-sheet region than any other force field. Similar to what was observed in the DSSP-PPII analysis, A99SB-disp and C36IDPSFF were found to have larger sampling in the α-helical regions than the other two force fields.

**Figure 6:**
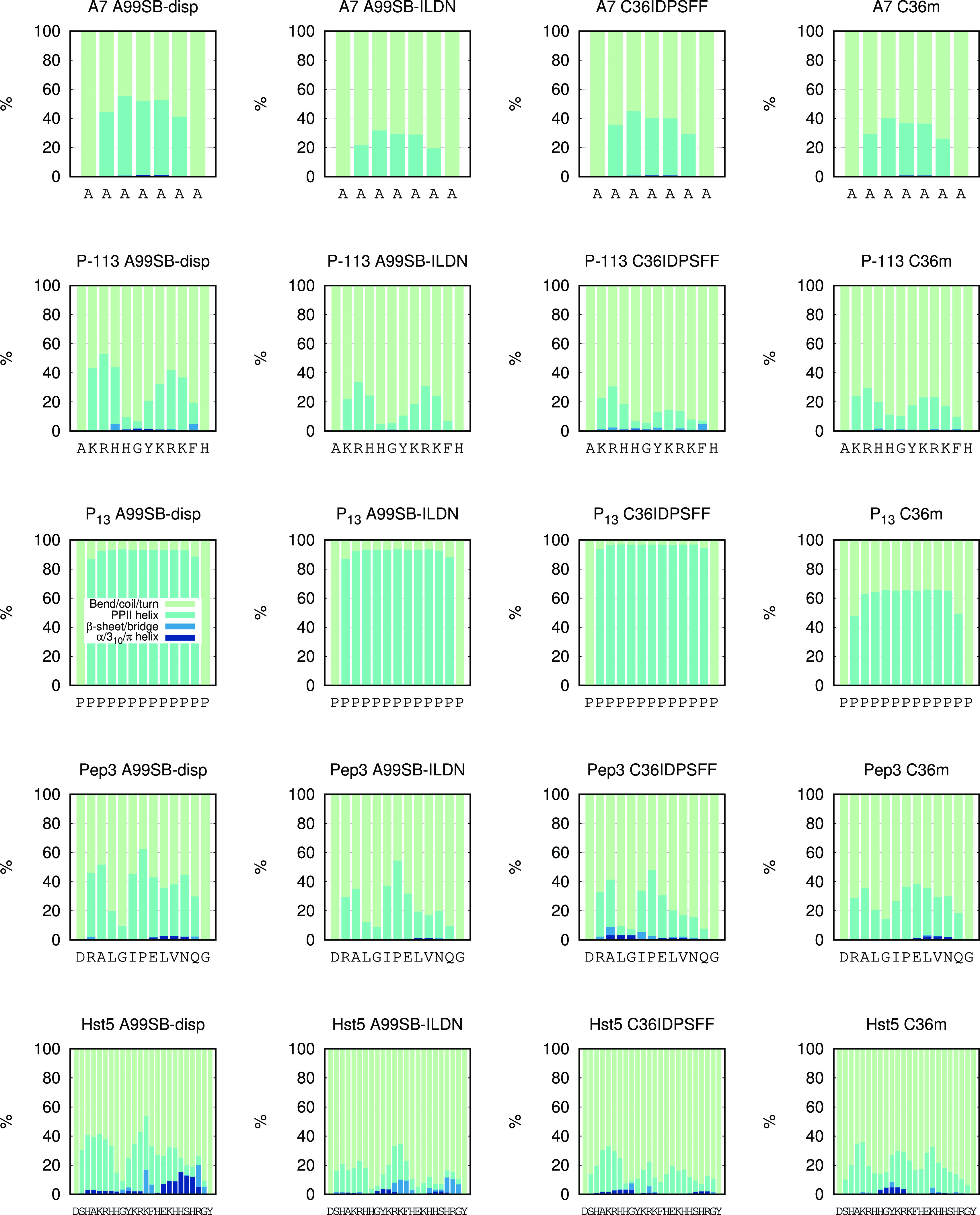
Stacked secondary structure histograms per amino acid residue of simulated peptides as obtained from DSSP-PPII algorithm.

Each simulated peptide was investigated with DSSP-PPII to identify the average secondary structure content per amino acid residue (Figure 6). All force fields gave similar secondary structure profiles for A7 and P-113, although a small difference was observed between the Amber and the CHARMM force fields. In P-113 the largest PPII content was centered around Arg-3 and Arg-9, and the lowest PPII content was found around Gly-6. The secondary structure profiles of P_13_ were similar for all force fields except for C36IDPSFF, where the PPII content was lower. For the Pep3 simulations, the largest PPII content was centered around Ala-3 and Pro-7 for all force fields. A small increase in PPII content was also observed around Asn-11 in Pep3 for all force fields except C36IDPSFF. The PPII content followed a sharper decrease toward the C-terminus in the simulations with A99SB-ILDN and C36IDPSFF, and the lowest PPII content was centered around Gly-5. The α/3_10_/π-helical content in Pep3 was found mainly around Val-10 except for in the C36IDPSFF simulation, where a larger portion was centered around Lys-4. For Hst5, the largest PPII content was found around Lys-5 and Lys-13 for all force fields, in addition to a smaller peak around Lys-17. A99SB-ILDN and C36m had their α/3_10_/π-helical content around Tyr-10, whereas it was located closer to the termini in the C36IDPSFF simulations and around His-19 in the A99SB-disp simulations. The simulation of Hst5 with A99SB-disp also gave rise to a low amount of α/3_10_/π-helical content throughout most of the peptide. The β-sheet/bridge content in Hst5 was found around Lys-13 and Arg-22 in the Amber simulations but was more randomly distributed in the CHARMM simulations.

Differences between the force fields were further analyzed for Hst5. Cluster analysis was performed for each individual force field, as well as for a concatenated trajectory in which all four force fields were included. The analysis was done using a root-mean-square-deviation (RMSD) cutoff of 0.5 nm. This value was chosen by examining the total population of the eight first clusters and varying the cluster radius (in steps of 0.05 nm) until their total population was closest to 50%. Additionally, using the same cutoff for all force fields made it easier to compare them. We do, however, note that because clustering methods compare “central structures”, they are not optimal to use for analyzing flexible peptides. Therefore, the number of clusters and their sizes that are presented here are not representations of the “truth”—they are simply used to compare the conformational sampling of the force fields.

**Figure 7:**
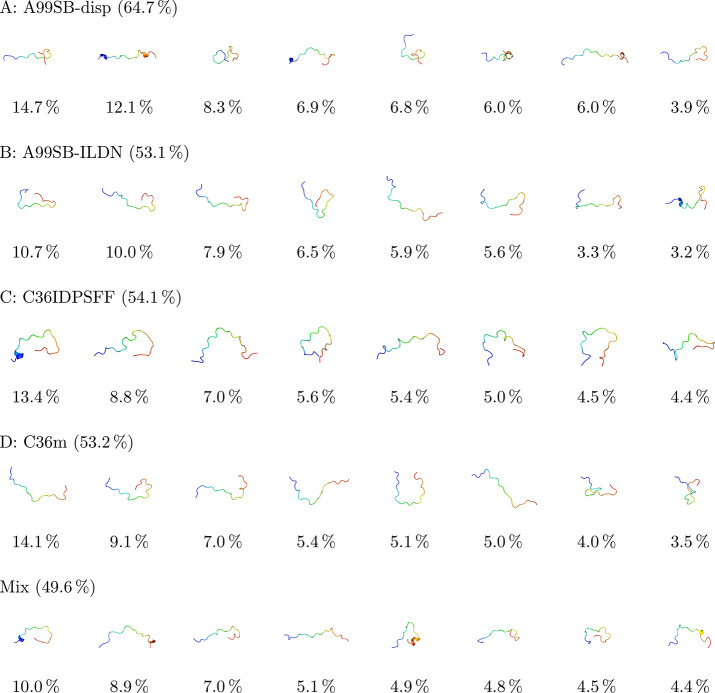
Representative structures of top eight cluster conformations of Hst5 as simulated with the four different force fields (A–D), as well as from a mixture of the four force fields (Mix). The total percentages of the top eight clusters are given above the structures, and the relative size of each individual cluster is given below each structure.

The representative structures of the top eight conformational clusters of Hst5 with the four force fields, as well as the force field mix, are presented in Figure 7. Visual inspection of the representative structures immediately reveals that the first cluster conformers are different for the different force fields. Comparing the combined percentage sizes of the top eight conformation clusters gives some indication of the relative variability of the conformations sampled by the four different force fields. A higher value means that there are fewer conformations sampled in the remaining clusters, which suggests a lower degree of conformational variability. By this reasoning, of the four selected force fields, A99SB-disp provides the smallest amount of conformational variability with the remaining three force fields being comparable.

**Figure 8:**
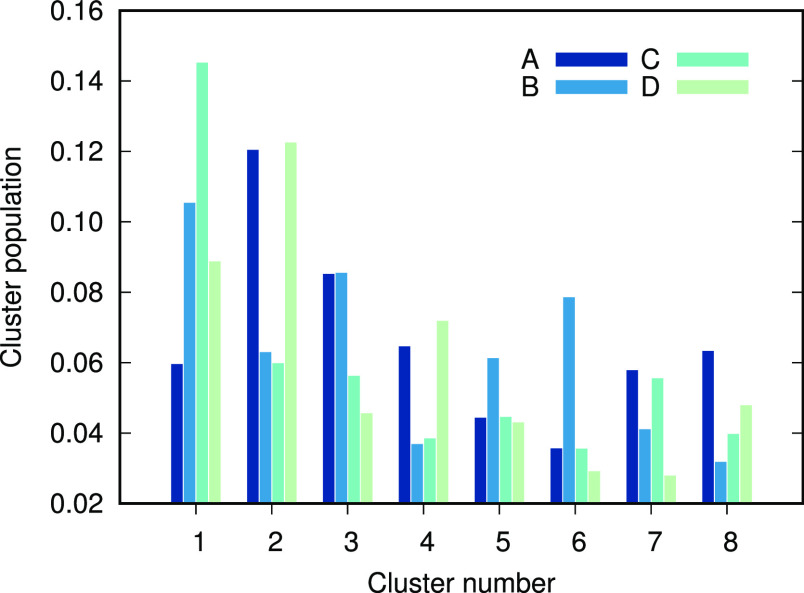
Weighted cluster population from the individual force fields in the top eight clusters of Hst5.

To get a more quantitative comparison among the force fields, their trajectories were concatenated, followed by a new cluster analysis where each structure could be traced back to its individual force field. The relative cluster populations of the individual force fields in the top eight clusters are illustrated in Figure 8. Although all force fields are represented in each cluster, they are not evenly distributed. For example, the first cluster is dominated by C36IDPSFF, whereas the second cluster mainly contains conformations from A99SB-disp and C36m. The fifth cluster is the most evenly distributed cluster across the force fields, and the sixth cluster is heavily dominated by A99SB-ILDN. From this analysis it is safe to say that, although the average properties of different force fields may be similar, the force fields’ individual conformational ensembles are rather different, which naturally leads to different secondary structure contents.

### 3.2 CD Prediction Using SESCA

**Figure 9:**
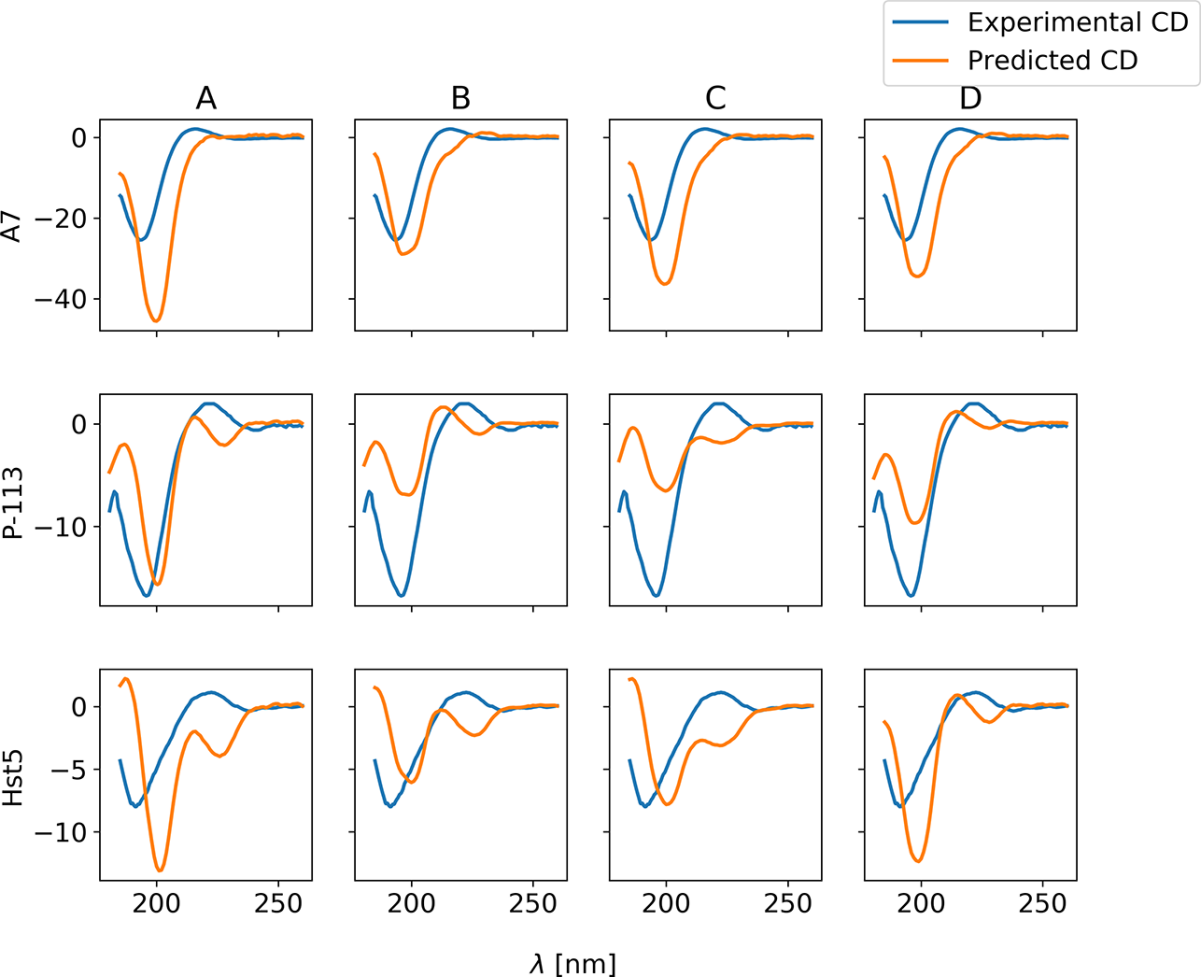
Comparison of experimental CD spectra with the ones predicted with SESCA from the conformational ensembles produced employing different force fields. The *y*-axes show the ellipticity, θ (deg cm^2^/dmol).

Since the four force fields give rise to different conformational ensembles, one may reasonably ask which of the force fields is more representative of the real conformational ensemble in solution. To answer this question, we may attempt to compare the simulations to experimental data. This ideally requires a forward model to predict the experimental observables from an ensemble of structures. We here used data from CD spectroscopy, as CD is highly sensitive of secondary structure composition, and we used SESCA^59^ as a forward model. Experimental data for A7, P-113, and Hst5 were obtained from Graf et al.,^63^ Han et al.,^70^ and Jephthah et al.,^48^ respectively. Unfortunately, and as also noted for other IDPs in the papers by Fagerberg et al.^71^ and Gopal et al.,^72^ it was not possible to obtain a meaningful agreement between the experimental CD spectra and those predicted by SESCA (Figure 9). This can be due to the fact that the main negative signature peak of a PPII helix may appear in experiments between 190 and 210 nm^73−75^ because of non-secondary-structure contributions, while the spectral contribution associated with a PPII conformation in SESCA has a fixed position. Also, given the relative scarcity of PPII structure in folded proteins, it may be difficult to deconvolute its contribution when developing prediction methods for CD spectra. Also, a qualitative analysis based on the intensity of the main negative peak does not provide a decisive suggestion of what force field may be the most reliable. This is also complicated by some intensity scaling that may be needed to take into account uncertainty in the estimate of the concentration of the sample used for the experimental CD data. At this stage it is not clear if the source of the problem may be the force fields’ inaccuracy, finite sampling, or the inaccuracy of the CD calculation for these kinds of systems.

### 3.3 Comparing A7 Simulations to Experimental NMR Scalar Couplings

A perhaps more accurate way of comparing simulations and experiments of flexible proteins and peptides is by investigating NMR scalar couplings. Since experimental *J*-coupling constants are available in the literature for A7^63^ and *J*-couplings are more commonly used in comparison with computational models, we decided to calculate *J*-couplings from our A7 simulations (see Methods). Simulations with A99SB-disp show the best agreement with the experimental data, followed by C36IDPSFF, while C36m and A99SB-ILDN give rise to a less good fit with the experimental data (Table 5). This result may in part reflect that the target data for optimizing A99SB-disp included the experimental *J*-couplings for Ala5.^38^ With 39 scalar couplings used to calculate the χ^2^ values, it appears that the deviations observed in C36m and A99SB-ILDN are greater than what would be expected by chance.

**Table 5:** Result of Comparison between the Experimental *J*-Coupling Constants of A7 and Those Calculated from the Conformational Ensembles Resulting from the Different Force Fields

FF	χ^2^
A	19.8
B	98.1
C	39.6
D	53.1

**Figure 10:**
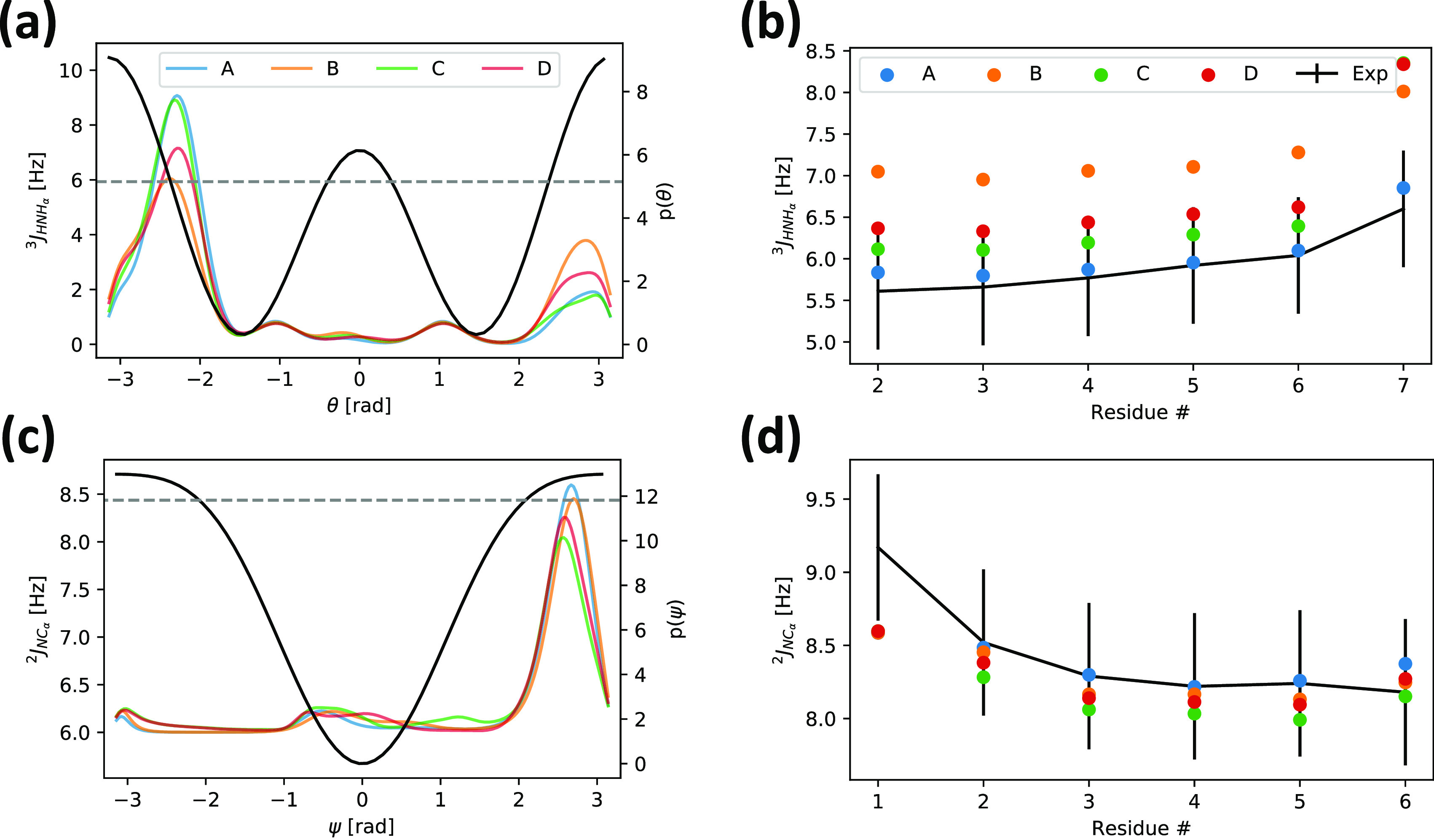
Overview of two *J*-coupling constants predicted from A7 simulations. (a) ^3^*J*_HNH_α__ and (c) ^2^*J*_NC_α__ as a function of the underlying angles are represented as solid black lines (left *y*-axes), the experimental couplings (average over residues) are represented as dashed gray lines (left *y*-axes), and the dihedral angle distributions from the different force fields are represented in different colors (right *y*-axes). (b) and (d) show the experimental couplings (with errors used for the calculation of the χ^2^ associated with the parametrizations of the Karplus relationship) as solid black lines, and the predicted couplings from the different force fields are represented with different colors.

We examined in more detail the ^3^*J*_HNH_α__ and ^2^*J*_NC_α__ couplings, as ^3^*J*_HNH_α__ can help discriminate the ϕ dihedral regions of β and α_r_/PPII elements, and ^2^*J*_NC_α__ can help discriminate the ψ dihedral regions of β/PPII and α_r_.^63^ We observe that the ψ angle distributions are relatively similar, with a strong peak corresponding to the β/PPII region, and the agreement with ^2^*J*_NC_α__ is evenly good (Figure 10c,d). More differences can be observed for ^3^*J*_HNH_α__, where especially A99SB-ILDN and C36m show a lower population in the α_r_/PPII (4–7 Hz^76^) and a higher population in an unclassified region, resulting in a lower agreement with the experimental ^3^*J*_HNH_α__ (Figure 10a,b).

### 3.4 Effect of Proline Residue Content

**Figure 11:**
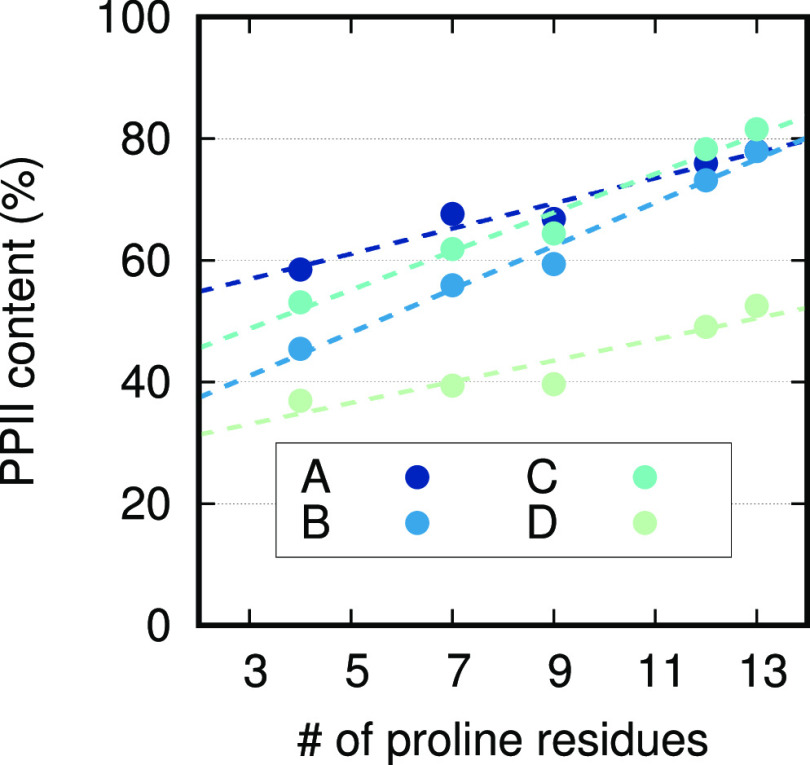
PPII helix content as obtained from DSSP-PPII analysis as a function of total number of Pro residues. Values from simulations of P_13_, V1, V2, V3, and V4, were used for this investigation.

A few variants of P_13_, V1–V4 (see Table 1), were investigated to see how the Pro content affected the PPII propensities, as estimated by how much the residues sampled the PPII regions of the Ramachandran maps. Figure 11 shows the PPII content as a function of the number of Pro residues in P_13_ and the peptide variants. All force fields yielded significant correlation (*p* < 0.05, see Table 6) between the PPII content and the number of Pro residues for P_13_ and the chosen variants, where an increased number of Pro residues provided a larger PPII content. The slopes of these trends in the linear regression, however, differ depending on what force field was used, with C36m having the smallest increase and A99SB-ILDN having the largest increase. Furthermore, A99SB-ILDN was the force field that provided the strongest correlation (see Table 6).

**Table 6:** Linear Regression Statistics of Figure 11^a^

	*m*	*c*	*r*^2^	*p*
A	2.076	50.691	0.948	0.005
B	3.561	30.322	0.983	0.001
C	3.181	39.218	0.973	0.002
D	1.738	27.875	0.870	0.021

a Slope (*m*), intercept (*c*), coefficient of determination (*r*^2^), and probability value (*p*).

**Figure 12:**
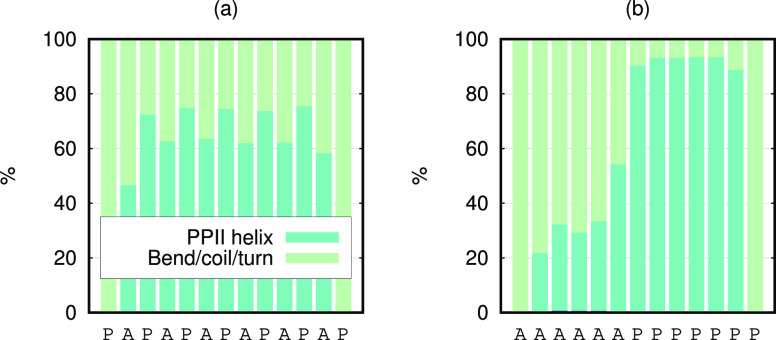
Stacked secondary structure histograms per amino acid residue of (a) V2 and (b) V2.2 as obtained from the DSSP-PPII algorithm.

V2.2 (see Table 1) was simulated using the A99SB-ILDN force field to investigate if the PPII content is affected by the relative position of the Pro residues in the amino acid sequence. The average PPII contents of V2 and V2.2 from the DSSP-PPII analysis were found to be essentially the same: 56 and 55%, respectively. Although the PPII content at first did not seem to be affected by the patterning of the Pro residues, histograms of the secondary structure per amino acid residue of V2 and V2.2 (Figure 12) revealed that the PPII content is significantly more localized to the Pro residues in V2.2, whereas it was more evenly distributed in V2. However, further investigation is needed to fully characterize this trend. It is, for example, necessary to study the effect of patterning of Pro in the other P_13_ variants as well. It would also be of interest to see how the trend is affected by the peptide length.

## 4 Conclusions

In this study we have evaluated the differences among four different force fields in simulations of five short peptides with varying PPII propensities. All force fields gave similar ensemble averages of the radius of gyration, although the averages by the C36IDPSFF force field were generally slightly smaller compared to the other force fields. All force fields appeared to sample comparable regions of conformational spaces (as probed by the first two principal components) for each individual peptide, although with slightly different probabilities. Similarly, all force fields sampled the PPII structure, but to different extents. Additionally, some force fields were more prone to sampling other secondary structure elements. For example, A99SB-disp and C36IDPSFF sampled more α/3_10_/π-helical and β-sheet/bridge content, C36m sampled less structured content than the other force fields, and A99SB-disp often had the highest PPII content. Direct comparison by conformational clustering revealed that the force fields have a bias toward different conformational clusters. CD prediction using SESCA was performed to examine which force field provided a more accurate conformational ensemble. Unfortunately, the method was not able to match the predicted and experimental spectra. We also calculated scalar couplings and compared them to experimental results for A7 and found two force fields (A99SB-disp and C36IDPSFF) that gave agreement roughly within expected error. We note that the calculations of both CD and scalar couplings contain contributions from all types of local structures and do not solely report on the accuracy of the PPII content. Finally, we investigated the effect of Pro residue content on the PPII content of short peptides containing only Ala and Pro, and we observed a correlation between the number of Pro residues in the amino acid sequence and the PPII content.

We conclude by highlighting that we need better methods to calculate experimental observables that are sensitive to secondary structure preferences for flexible peptides. Such methods are often parametrized using folded protein structures and, thus, may be difficult to apply or inaccurate for disordered peptides and proteins.^77^ In particular, we stress the need for better methods to link populations of PPII-like structures in simulations to a broader range of experimental observables and note that NMR chemical shifts can also be used for this purpose.^78,79^ This is especially needed for simulations of proteins in which PPII might have a significant role, such as for example Hst5, SH3-binding peptides, and collagen.

## Supporting Information Available

Probability distributions, autocorrelation functions, block average error estimates, and ω angle as a function of time (PDF)
