# Supplementary material for: Force Field Effects in Simulations of Flexible Peptides with Varying Polyproline II Propensity
Source: J Chem Theory Comput. 2021 Sep 15;17(10):6634–46. doi: 10.1021/acs.jctc.1c00408 (PMC8515809; doi:10.1021/acs.jctc.1c00408)
Supplement: Supplementary file 1 — ct1c00408_si_001.pdf [file ct1c00408_si_001.pdf]

Supporting information for: Force field effects in simulations of flexible peptides with varying polyproline II propensity

Stéphanie Jephthah,[†] Francesco Pesce,[‡] Kresten Lindorff-Larsen,[*,‡] and Marie Skepö[*,†]

†*Division of Theoretical Chemistry, Lund University, Lund, Sweden*

‡*Structural Biology and NMR Laboratory & the Linderstrøm-Lang Centre for Protein Science, Department of Biology, University of Copenhagen, Copenhagen, Denmark*

E-mail: lindorff@bio.ku.dk; marie.skepo@teokem.lu.se

Phone: +45 35 32 20 27; +46 46 222 33 66

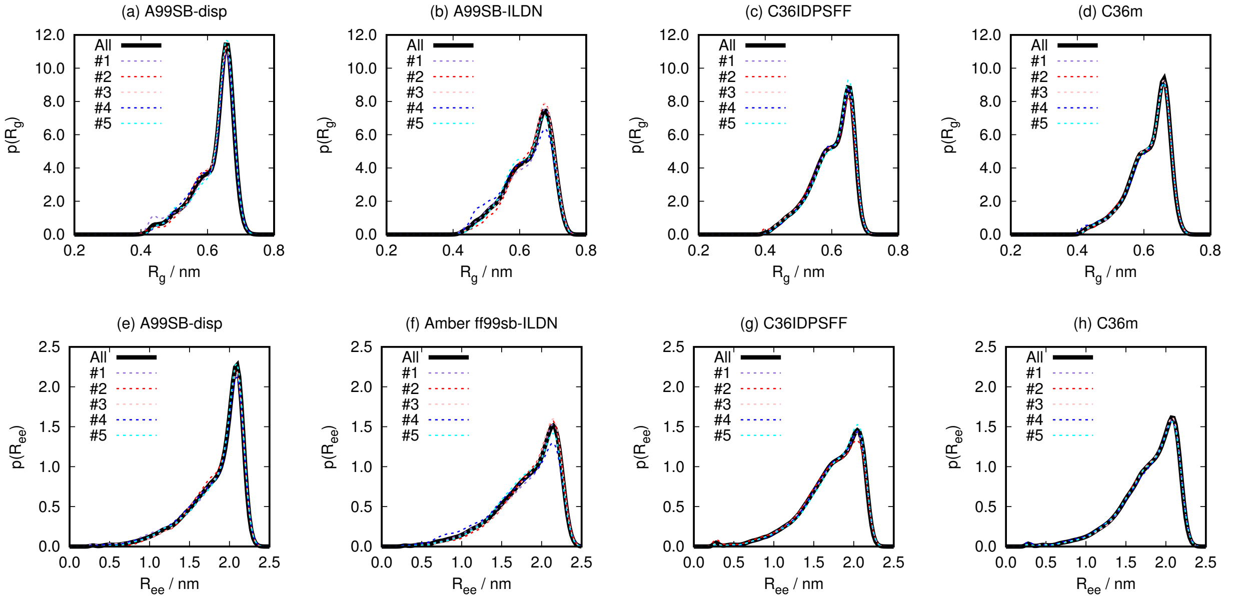

Figure S1: Probability distributions of the radius of gyration, R_g (top row), and the end-to-end distance, R_{ee} (bottom row), of A7 as obtained from the four different force fields.

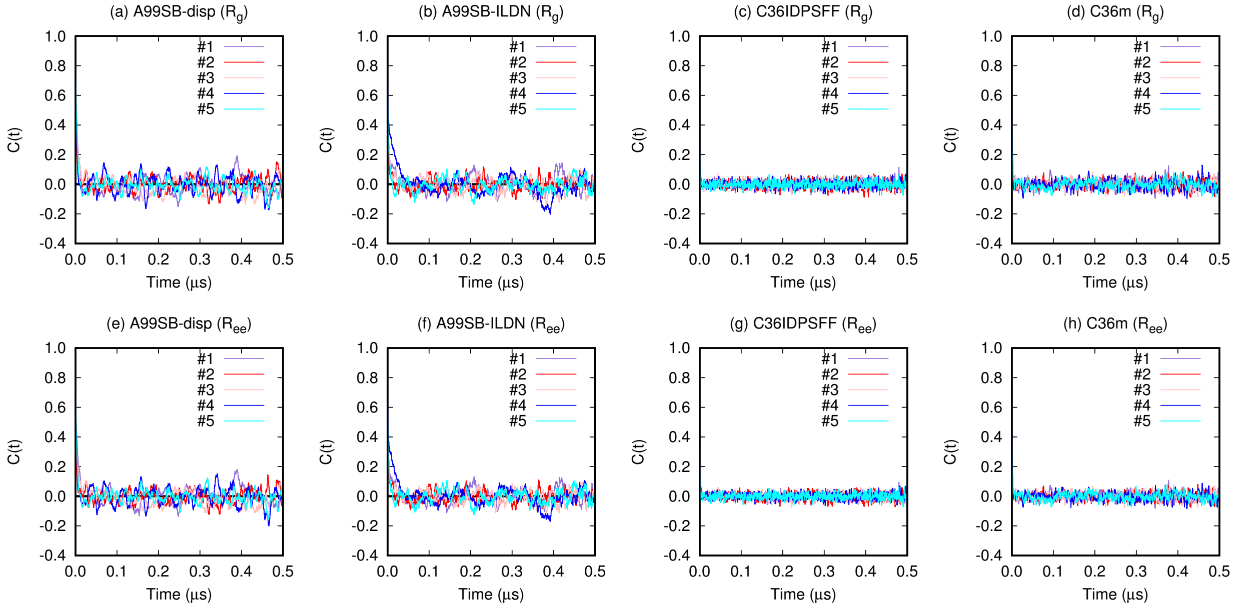

Figure S2: Auto-correlation functions of the radius of gyration, R_g (top row), and the end-to-end distance, R_{ee} (bottom row), of A7 as obtained from the four different force fields.

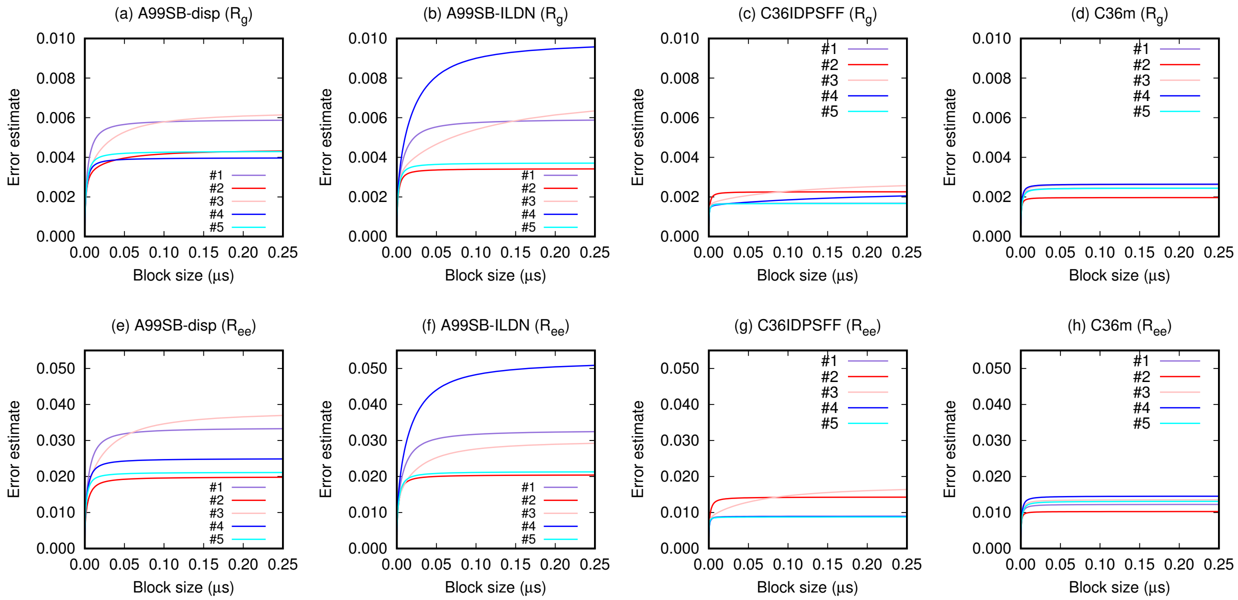

Figure S3: Block average error estimates of the radius of gyration, R_g (top row), and the end-to-end distance, R_{ee} (bottom row), of A7 as obtained from the four different force fields.

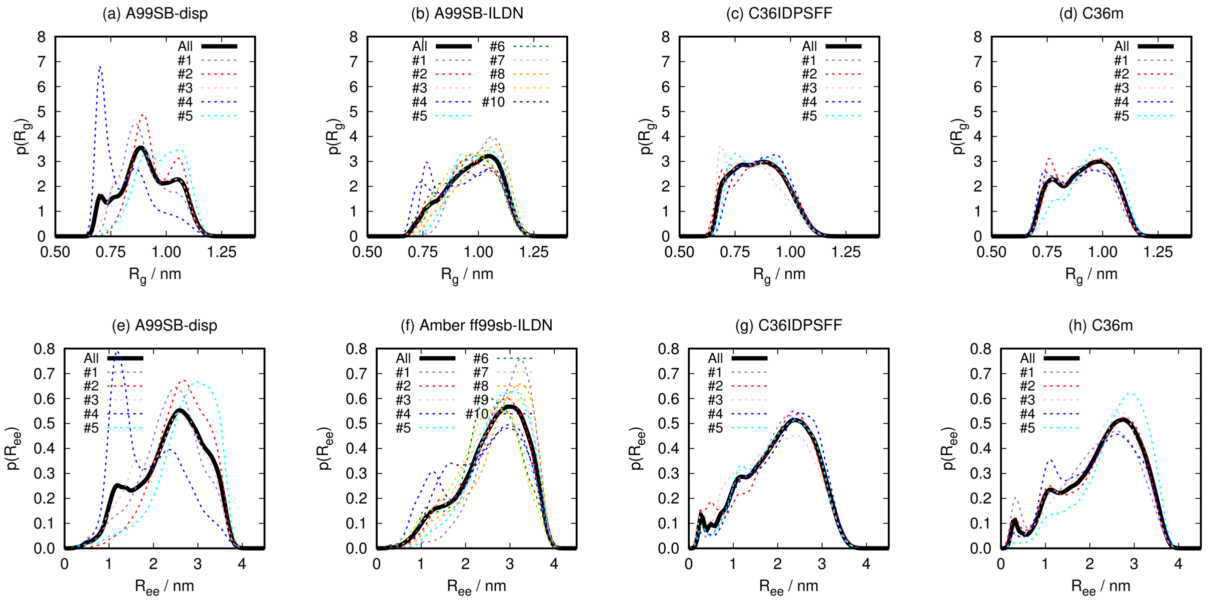

Figure S4: Probability distributions of the radius of gyration, R_g (top row), and the end-to-end distance, R_{ee} (bottom row), of P-113 as obtained from the four different force fields.

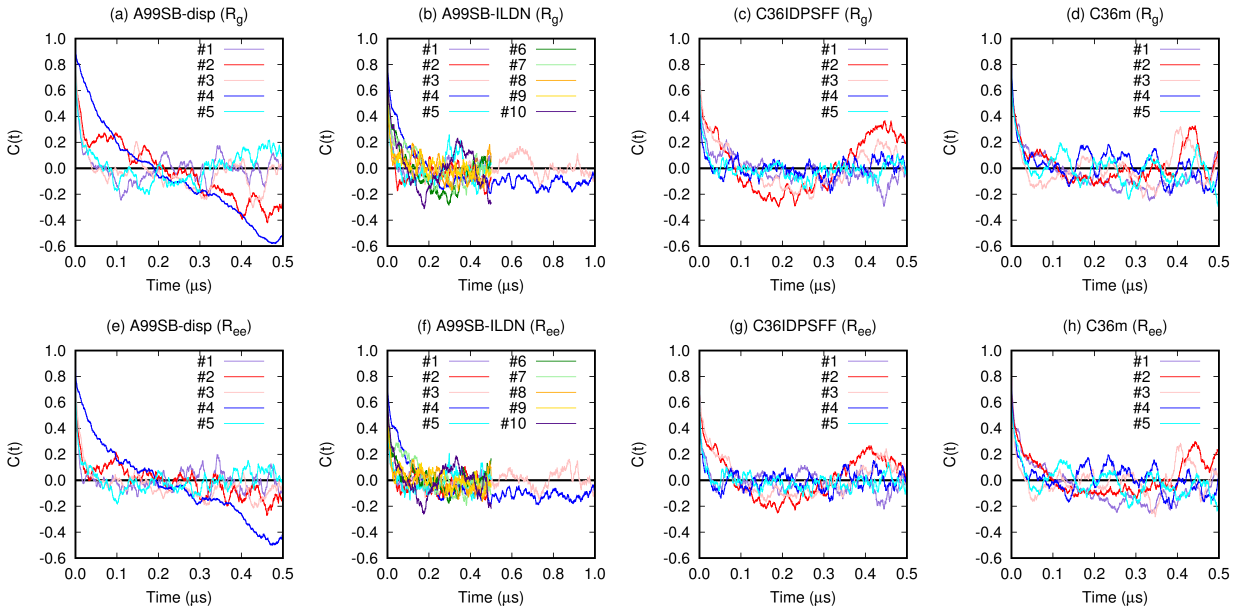

Figure S5: Auto-correlation functions of the radius of gyration, R_g (top row), and the end-to-end distance, R_{ee} (bottom row), of P-113 as obtained from the four different force fields.

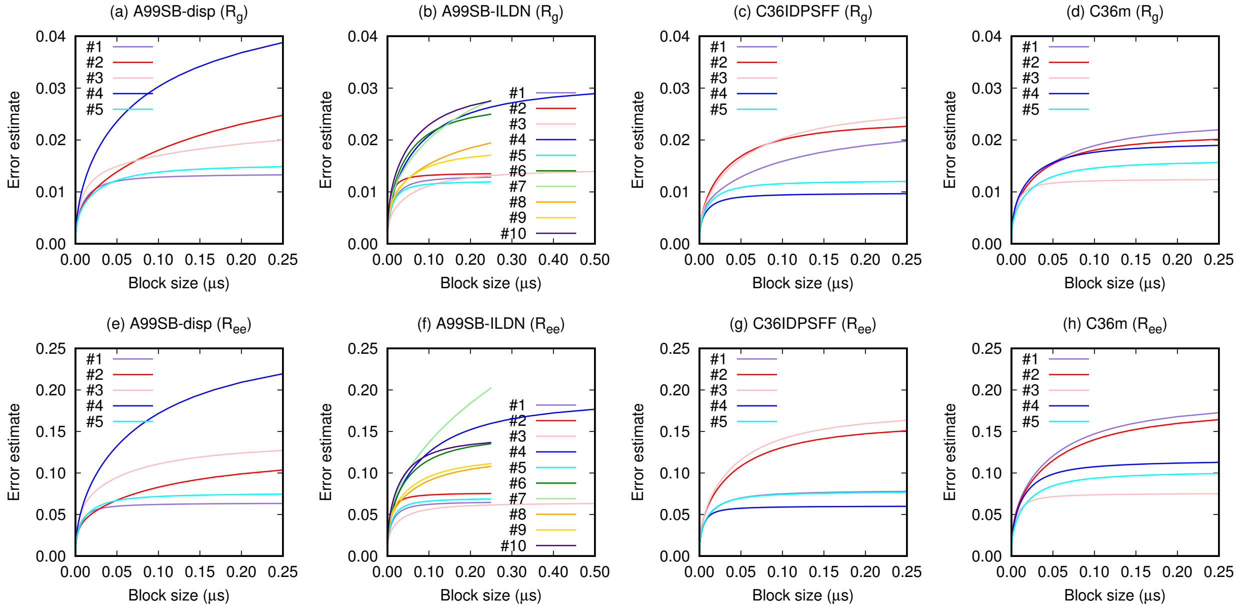

Figure S6: Block average error estimates of the radius of gyration, R_g (top row), and the end-to-end distance, R_{ee} (bottom row), of P-113 as obtained from the four different force fields.

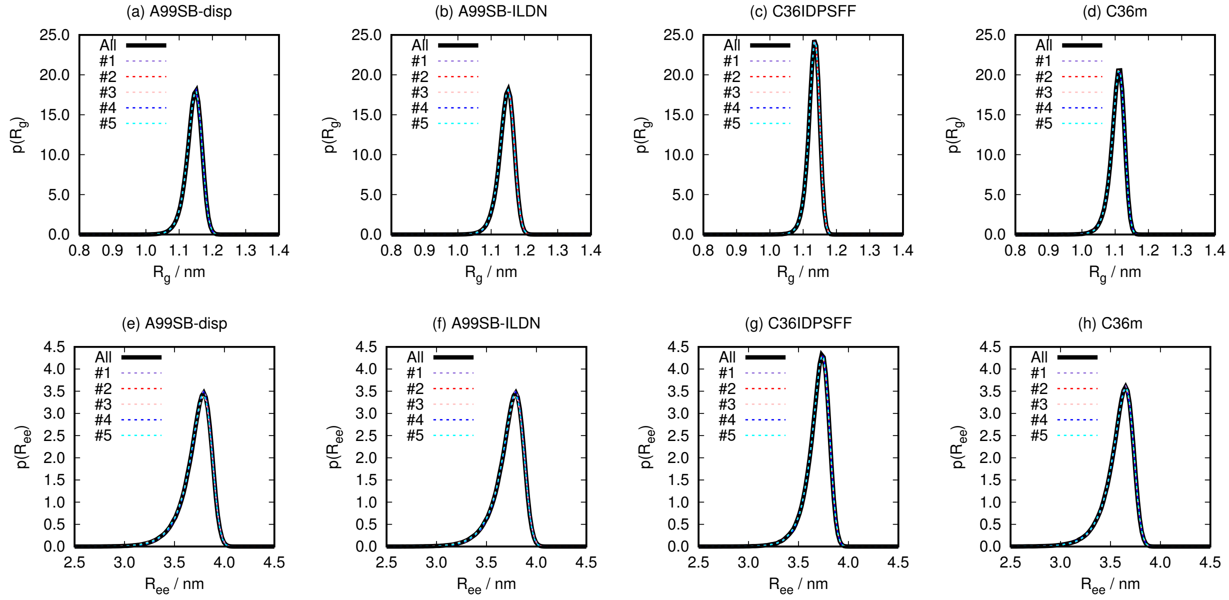

Figure S7: Probability distributions of the radius of gyration, R_g (top row), and the end-to-end distance, R_{ee} (bottom row), of P_{13} as obtained from the four different force fields.

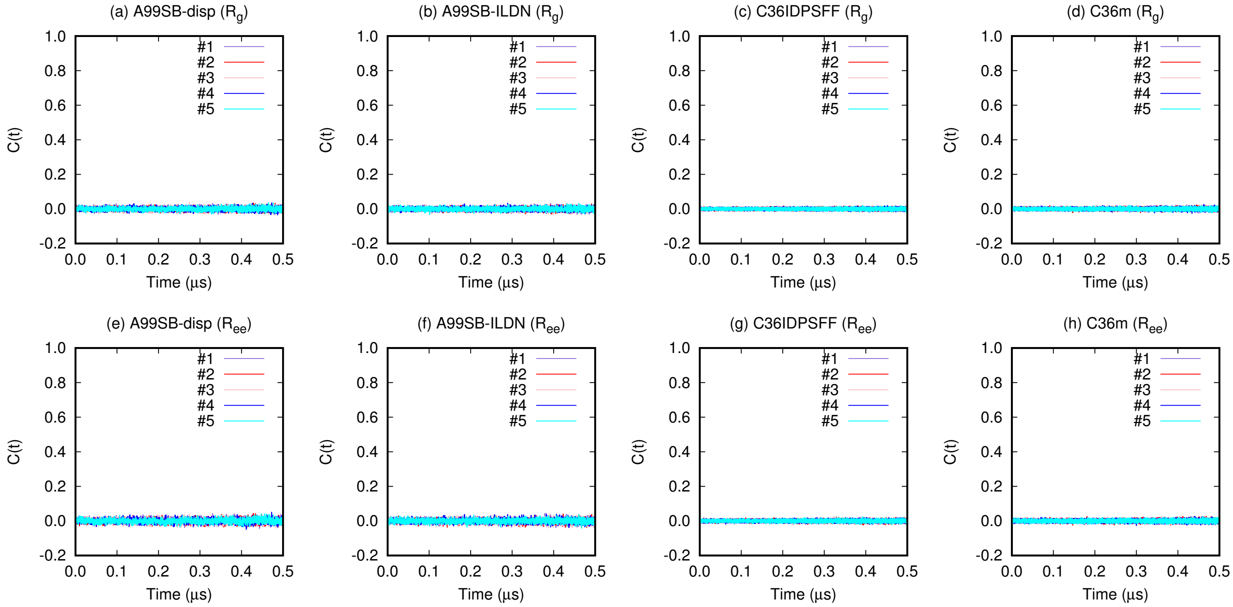

Figure S8: Auto-correlation functions of the radius of gyration, R_g (top row), and the end-to-end distance, R_{ee} (bottom row), of P_{13} as obtained from the four different force fields.

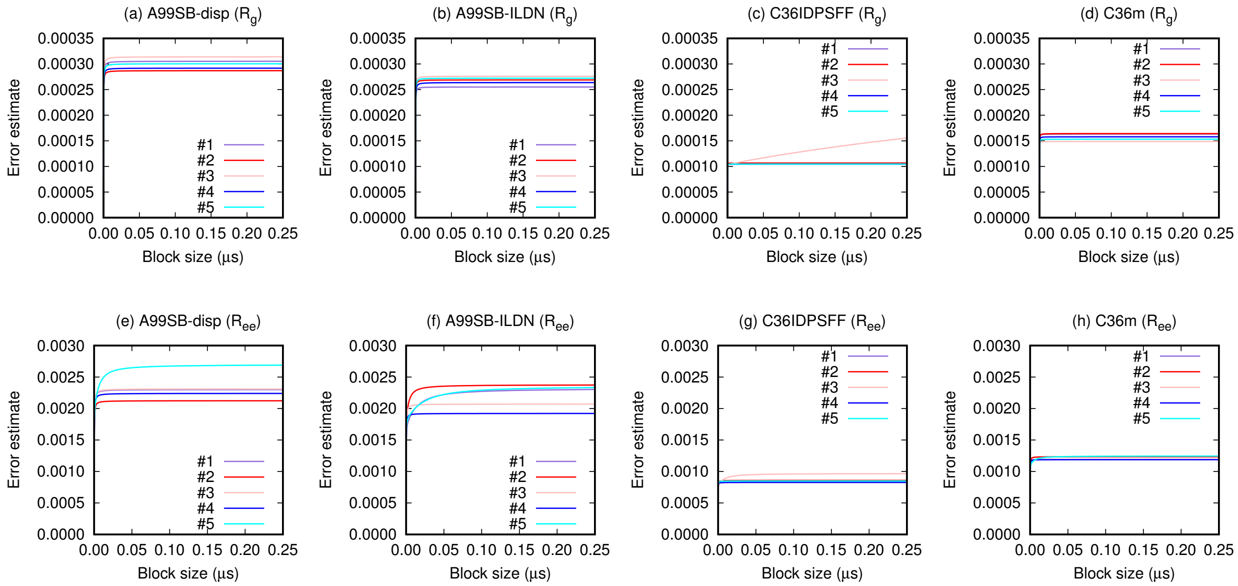

Figure S9: Block average error estimates of the radius of gyration, R_g (top row), and the end-to-end distance, R_{ee} (bottom row), of P_{13} as obtained from the four different force fields.

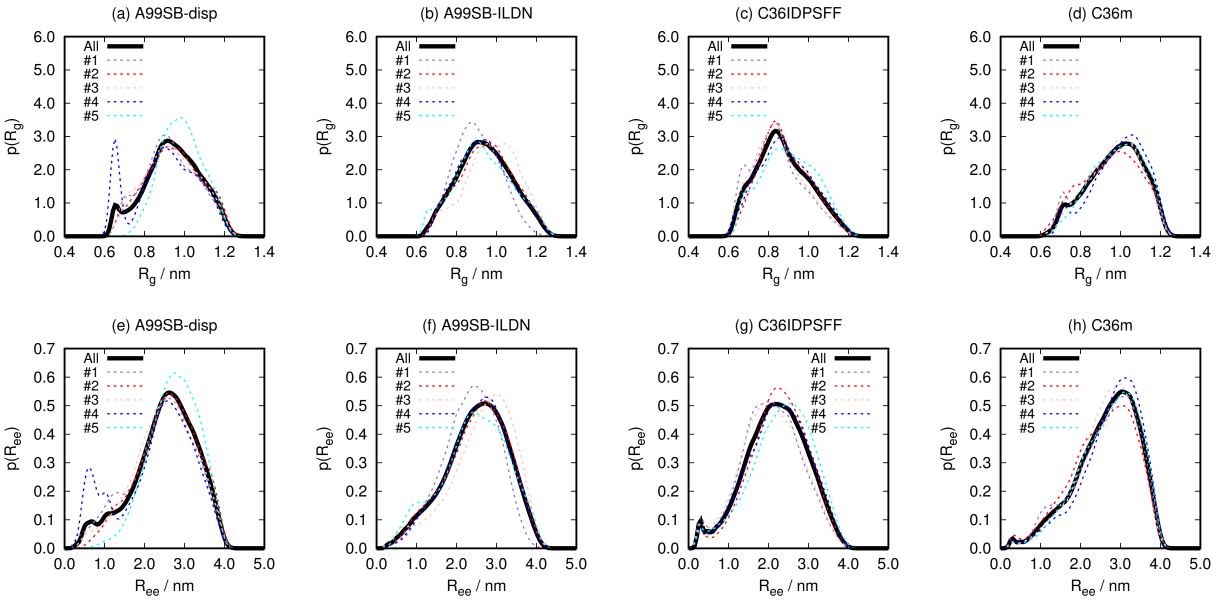

Figure S10: Probability distributions of the radius of gyration, R_g (top row), and the end-to-end distance, R_{ee} (bottom row), of Pep3 as obtained from the four different force fields.

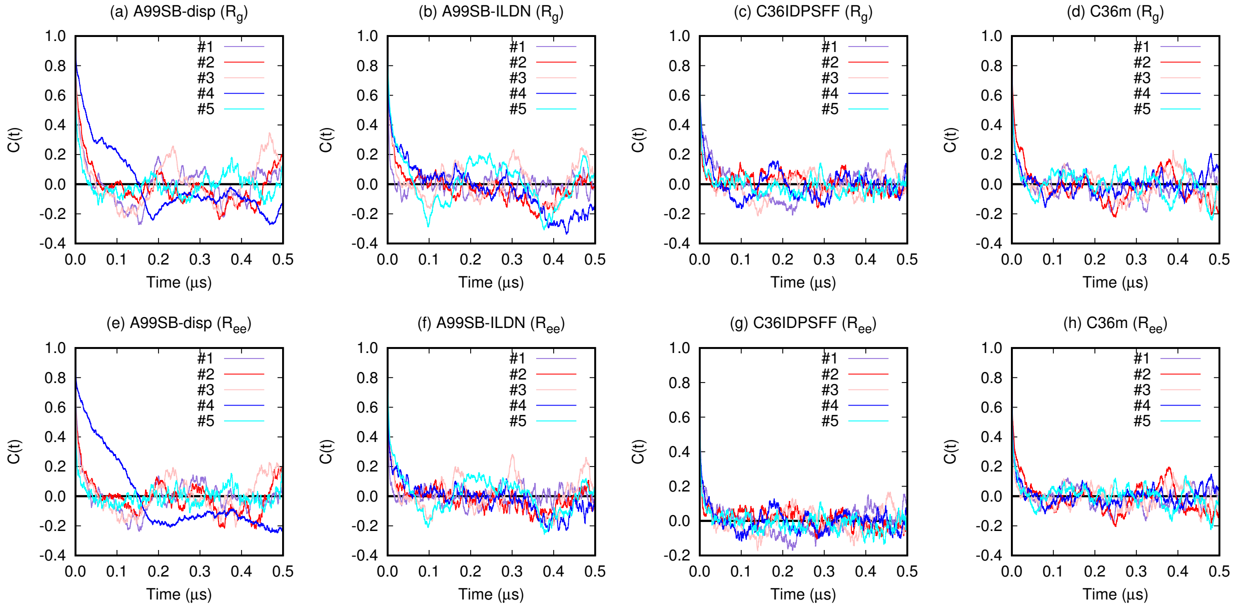

Figure S11: Auto-correlation functions of the radius of gyration, R_g (top row), and the end-to-end distance, R_{ee} (bottom row), of Pep3 as obtained from the four different force fields.

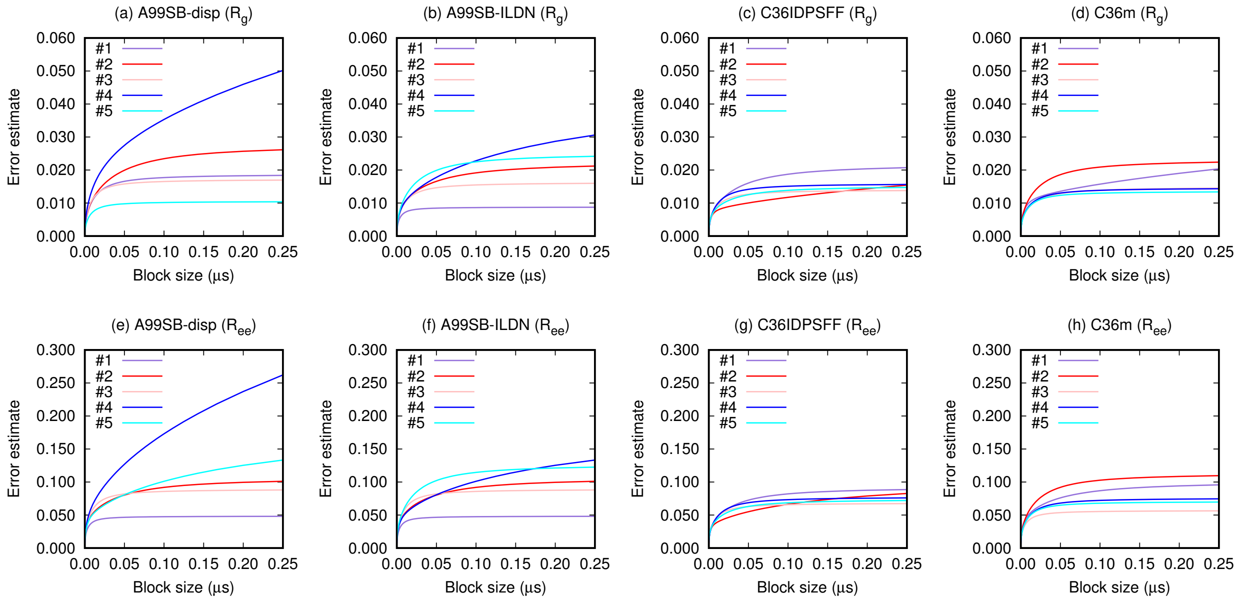

Figure S12: Block average error estimates of the radius of gyration, R_g (top row), and the end-to-end distance, R_{ee} (bottom row), of Pep3 as obtained from the four different force fields.

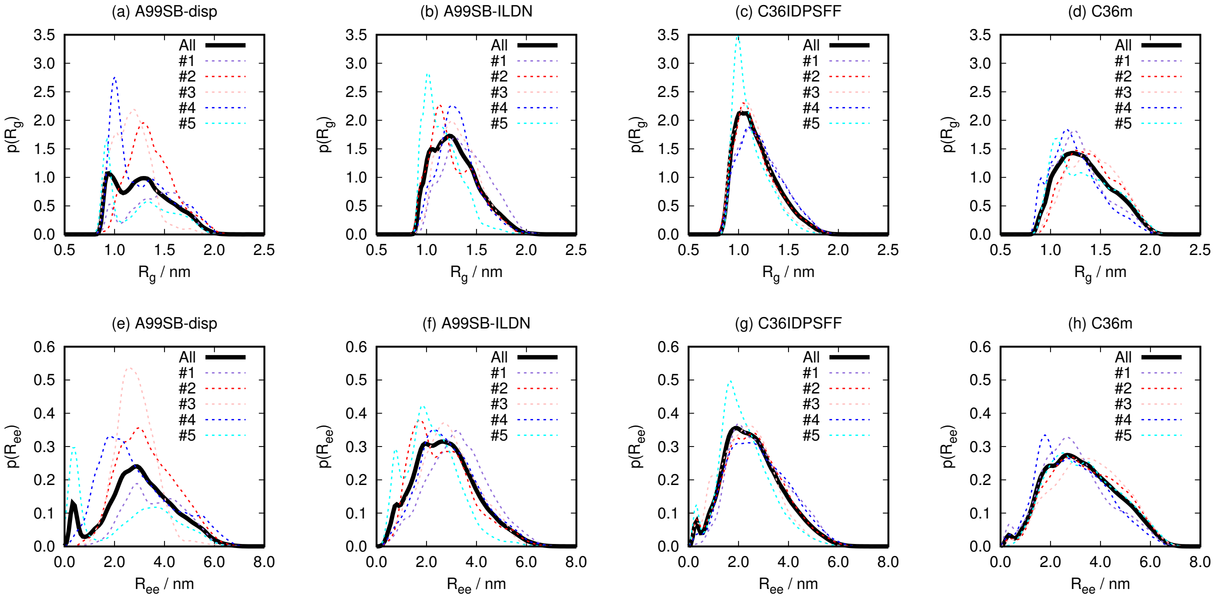

Figure S13: Probability distributions of the radius of gyration, R_g (top row), and the end-to-end distance, R_{ee} (bottom row), of Hst5 as obtained from the four different force fields.

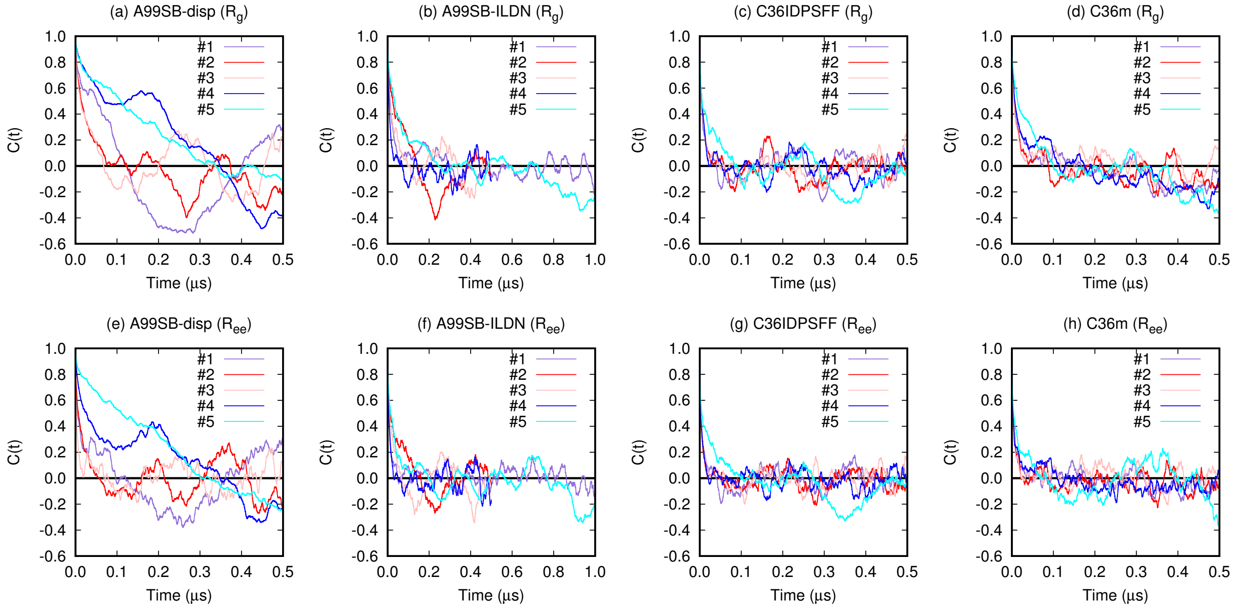

Figure S14: Auto-correlation functions of the radius of gyration, R_g (top row), and the end-to-end distance, R_{ee} (bottom row), of Hst5 as obtained from the four different force fields.

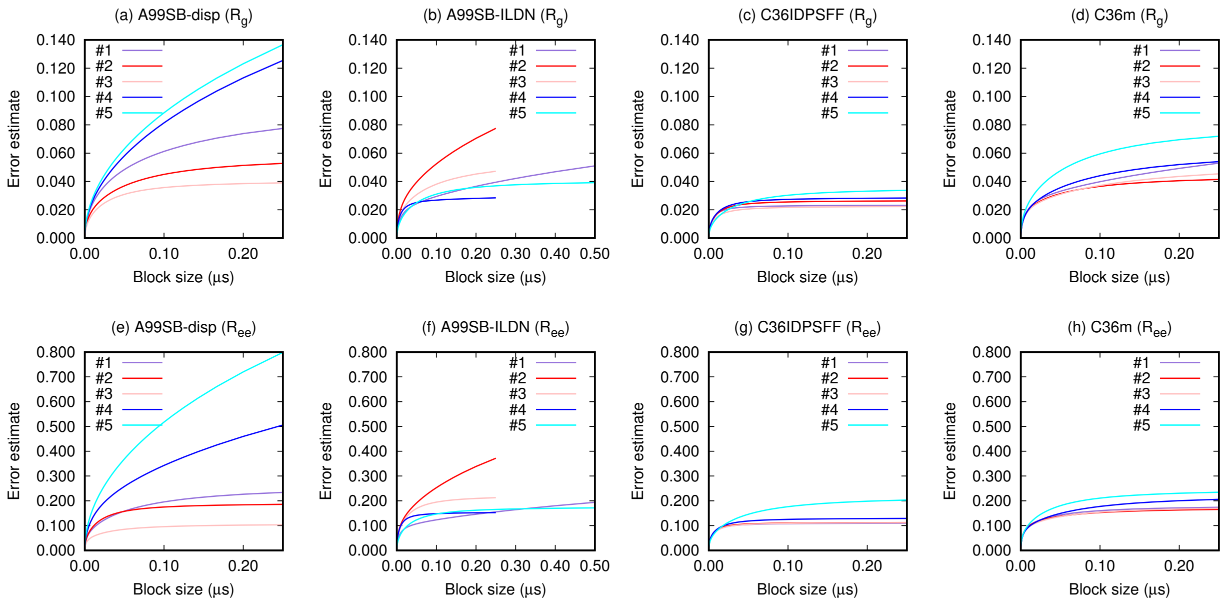

Figure S15: Block average error estimates of the radius of gyration, R_g (top row), and the end-to-end distance, R_{ee} (bottom row), of Hst5 as obtained from the four different force fields.

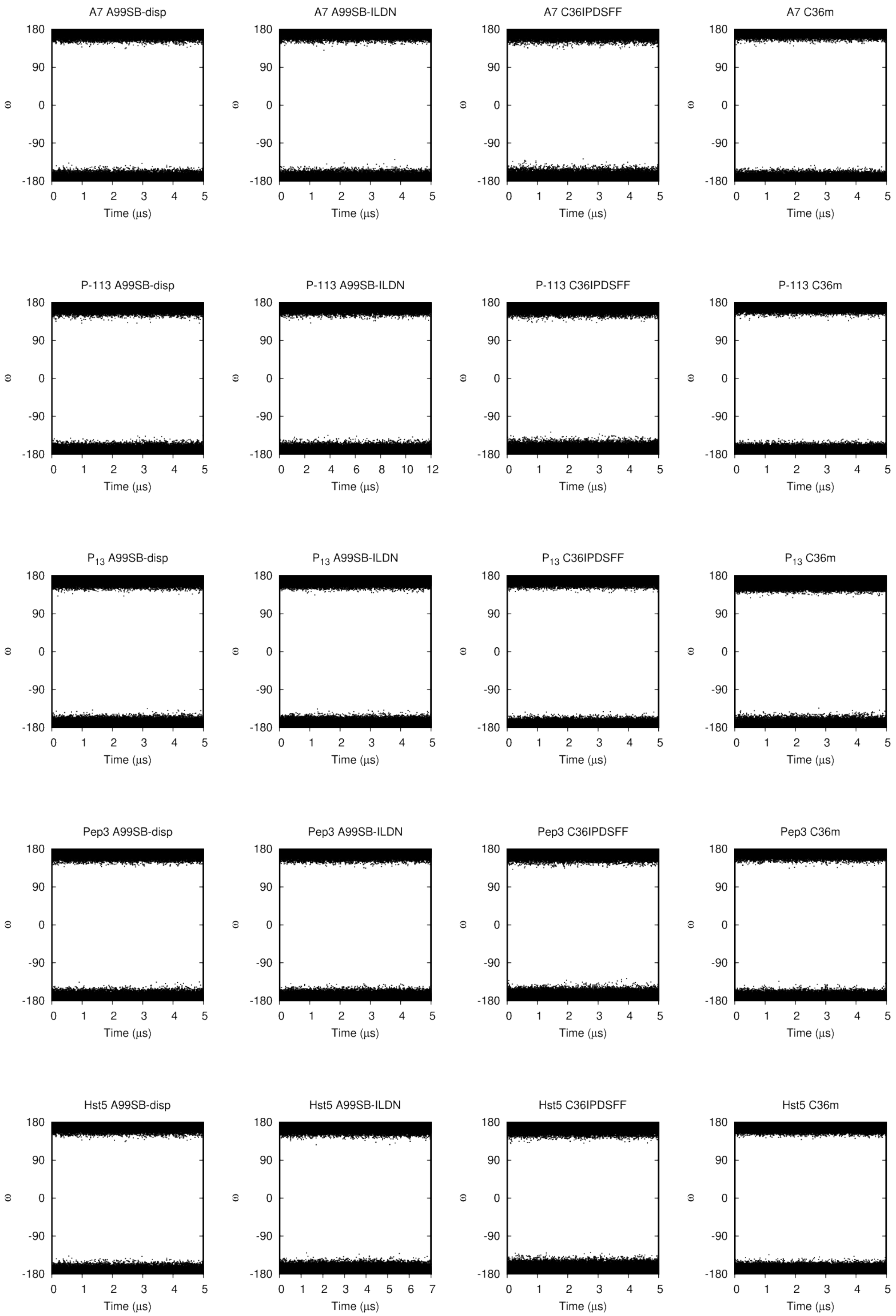

Figure S16: The ω angle as a function of simulation time for all simulations of the five main peptides and the four different force fields.
